# Rational engineering of a modular bacterial CRISPR–Cas activation platform with expanded target range

**DOI:** 10.1093/nar/gkab211

**Published:** 2021-04-06

**Authors:** Maria Claudia Villegas Kcam, Annette J Tsong, James Chappell

**Affiliations:** Department of BioSciences, Rice University, 6100 Main Street, MS 140, Houston, TX 77005, USA; Department of BioSciences, Rice University, 6100 Main Street, MS 140, Houston, TX 77005, USA; Department of BioSciences, Rice University, 6100 Main Street, MS 140, Houston, TX 77005, USA; Department of Bioengineering, Rice University, 6100 Main Street, MS 142, Houston, TX 77005, USA

## Abstract

CRISPR–Cas activator (CRISPRa) systems that selectively turn on transcription of a target gene are a potentially transformative technology for programming cellular function. While in eukaryotes versatile CRISPRa systems exist, in bacteria these systems suffer from a limited ability to activate different genes due to strict distance-dependent requirements of functional target binding sites, and require greater customization to optimize performance in different genetic and cellular contexts. To address this, we apply a rational protein engineering approach to create a new CRISPRa platform that is highly modular to allow for easy customization and has increased targeting flexibility through harnessing engineered Cas proteins. We first demonstrate that transcription activation domains can be recruited by CRISPR–Cas through noncovalent protein-protein interactions, which allows each component to be encoded on separate and easily interchangeable plasmid elements. We then exploit this modularity to rapidly screen a library of different activation domains, creating new systems with distinct regulatory properties. Furthermore, we demonstrate that by harnessing a library of circularly permuted Cas proteins, we can create CRISPRa systems that have different target binding site requirements, which together, allow for expanded target range.

## INTRODUCTION

Since their discovery, RNA-guided CRISPR–Cas ribonuclease systems have transformed many aspects of biological sciences, biotechnology and medicine (1–3). In particular, engineering of CRISPR–Cas systems has led to a powerful suite of *trans*-acting gene regulatory tools able to precisely program gene expression in a diversity of cellular contexts. Owing to the ease of designing and synthesizing guide RNAs (gRNAs) that determine target specificity, CRISPR–Cas regulators have found broad utility for performing targeted gene perturbation studies to uncover gene function (2,4–10) and connectivity (2,4–6,11,12), and to rapidly optimize strains for bioprocesses (13–20). Additionally, creation of orthogonal and composable CRISPR–Cas controlled promoter elements has enabled construction of genetic circuits able to perform cellular computations and signal processing (21–26).

In bacteria, the first iteration of CRISPR–Cas regulators was CRISPR interference (CRISPRi), which uses a nuclease-dead Cas9 (dCas9) and a single guide RNA (sgRNA) complex to sterically block transcription initiation and elongation (27,28). Extending this, further innovations have sought to use the CRISPR–dCas9 complex as a localization platform and to control gene expression through recruitment of protein effector domains. For example, CRISPR–Cas activators (CRISPRa) have been created by harnessing CRISPR–dCas9 systems to recruit protein activation domains (ADs) that stimulate transcription when localized in proximity to promoter elements. Different engineering approaches have been used to obtain CRISPRa systems, such as performing fusions of ADs directly to dCas9 (28,29) or engineering sgRNAs to recruit ADs (30).

While in eukaryotes CRISPRa systems have provided robust tools for activating transcription that have resulted in the widespread use of these technologies, in bacteria they have not had the same impact because of several technical limitations. First, it is increasingly clear that currently available ADs offer distinct regulatory properties and trade-offs, and different ADs are likely to be optimal depending on the exact gene target or application. For example, some bacterial ADs, such as those derived from the transcription factor SoxS (31,32) or RNAP subunits (28), show highest activation when localized immediately upstream (60–100 bp) of a target gene's transcription start site (TSS), while other ADs, such as those based on the AsiA anti-sigma factor (33), operate at longer distances (>100–190 bp upstream). ADs can also have different trade-offs between fold of activation and other performance criteria, such as off-target effects, growth inhibition and requirement of knockout strains (28,31,33). Specialized ADs are also required to activate promoter elements that utilize alternative sigma factors (34) or to allow creation of CRISPRa systems for different bacterial species (35,36). Unlike eukaryotic CRISPRa systems that have largely relied upon a handful of ADs (29,37,38) to achieve activation in diverse genetic and cellular contexts (37), bacterial CRISPRa systems likely require a greater repertoire of components and it remains unclear if an optimal AD has been identified. A second challenge of bacterial CRISPRa systems are distance-dependent activation patterns that significantly limit functional CRISPRa binding sites. Specifically, it was recently shown that bacterial CRISPRa systems that use sgRNA-based recruitment have activation patterns that are periodical, in which strong activation is only seen when the system is targeted within a 2–4 bp window that repeats every 10–11 bp from the TSS (32,34). This significantly limits the targeting range as only genes with correctly positioned upstream binding sites can be activated.

Bacterial CRISPRa systems represent a potentially transformative technology; however, work remains to be done to create a versatile tool. Towards this goal, we apply a rational protein engineering approach to create a new CRISPRa platform that is highly modular, allowing for facile exchange of new ADs, and presents expanded target range, through the exchange of different engineered dCas9 variants with different binding site requirements. First, recognizing that different ADs have distinct properties, we characterize a panel of ADs and identify the N-terminal domain of the alpha subunit of RNAP (αNTD) to robustly activate compared to previously reported ADs. Next, we explore modular approaches to recruit ADs to dCas9 through noncovalent protein-protein interaction sequences called SYNZIP domains (39,40). This modular approach presents comparable fold activation levels as using covalent linkers while allowing ADs to be encoded on separate and interchangeable plasmids, therefore allowing for facile exchange with dCas9-encoding plasmids. We exploit this enhanced modularity to rapidly characterize a panel of αNTD ADs derived from diverse bacterial species. Finally, to overcome the limitations in targeting range, we utilize circularly permuted variants of dCas9 (cpdCas9) that allow localization of ADs to distinct positions within the tertiary structure, and as a result, shift activation patterns. By screening a library of cpdCas9 variants, we identify novel CRISPRa systems that can activate from distinct positions compared to wild-type dCas9, thus expanding the number of targetable promoter elements. Taken together, our CRISPRa platform offers a high degree of modularity, in which plasmids encoding different dCas9 variants can be synergistically combined with different ADs to create unique combinations optimized for different gene targets and applications.

## MATERIALS AND METHODS

### Plasmid assembly

All plasmids used in this study can be found in Supplementary Table S1 with key sequences provided in Supplementary Table S2–S7. A schematic of representative plasmids used is shown in Supplementary Figure S1. dCas9 variants were expressed with a P_tet_, P_bad_ or a constitutive J23150 promoter in a p15a chloramphenicol resistant vector. sgRNAs were expressed with a J23119 promoter in a ColE1 ampicillin resistant vector. Constructs containing ADs fused to SYNZIP were expressed with J23106 promoter in a CloDF spectinomycin resistant vector. Reporter plasmids were cloned from pJF076Sa plasmid (addgene #113322) (31,32)—an RFP reporter with J23117 promoter and upstream NGG-rich sequence—into a kanamycin resistant pSC101 vector. Constitutive promoter sequences are available at the iGEM Registry of Standard Biological Parts (parts.igem.org). Plasmids were assembled through a combination of PCR ligations, Gibson assembly (41) or Golden Gate assembly (42).

### Fluorescence measurements

**Figure 1.**
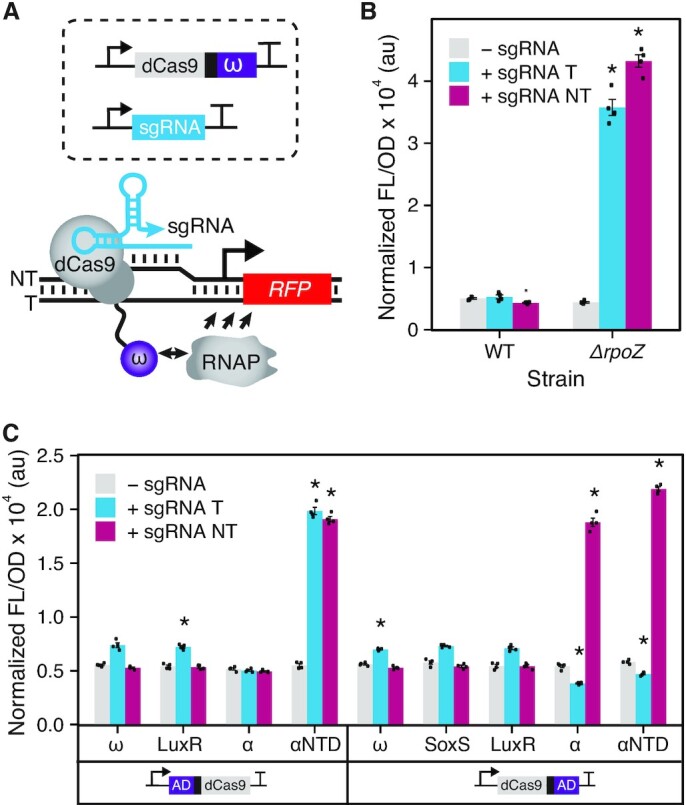
Synthetic transcription activation with dCas9 fusions to ADs. (**A**) Schematic of a CRISPRa system composed of dCas9 fused to the ω subunit of RNAP (dCas9-ω). For characterization, dCas9-ω in complex with the sgRNA was targeted upstream of a promoter driving RFP expression. Activation is achieved by local recruitment of RNAP to promoter elements. (**B**) Fluorescence characterization of a dCas9-ω system in wild-type strain *E*. *coli* K-12 BW25113 (WT) and a modified strain that lacks the gene encoding the ω subunit (*ΔrpoZ*). (**C**) Fluorescence characterization of dCas9 with N- or C-terminal fusions to different ADs through a two-alanine linker in *E. coli* MG1655 strain. ADs are derived from RNAP subunits: ω, α and αNTD; or transcription factors: SoxS and LuxR. Fluorescence measurements (measured in units of fluorescence [FL]/optical density [OD] at 600 nm) were performed with *E. coli* cells transformed with an RFP reporter plasmid, a plasmid encoding the dCas9 fusions, and a sgRNA-encoding plasmid or a no-sgRNA control plasmid. sgRNA variants used targeted PAMs located at 80 bp upstream of the promoter TSS on the template strand (+ sgRNA T) or 81 bp upstream on the non-template stand (+ sgRNA NT). Data represent mean values and error bars represent s.d. of *n* = 4 biological replicates. A two tailed Student's *t* test was used to calculate *P* value comparing against the no-sgRNA control. * *P* < 0.0001; *P* > 0.0001 has no asterisk.

Fluorescence characterization was performed using *Escherichia coli* strain MG1655, except for data shown in Figure 1B where *E. coli* K-12 BW25113 and *E. coli* K-12 BW25113 *ΔrpoZ* (JW3624-1, Keio collection) was used (43). Each experiment was performed with four biological replicates. For each condition, plasmid combinations were transformed into chemically competent *E. coli* cells and plated on LB + Agar (Difco) plates containing combinations of 100 μg ml^−1^ carbenicillin, 34 μg ml^−1^ chloramphenicol, 25 μg ml^−1^ kanamycin and 50 μg ml^−1^ spectinomycin, depending on the plasmids used, and incubated ∼17 h overnight at 37 °C. In some experiments involving transformation of ≥3 plasmids, a subset of plasmids was first transformed, competent cells prepared from resulting colonies and the remaining plasmid transformed. Plates were taken out of the incubator and left at room temperature for ∼7 h. Single colonies were used to inoculate four cultures of 300 μl of LB containing antibiotics at the concentrations described above in a 2 ml 96-well block (Costar). 96-well blocks were incubated overnight in a Vortemp 56 (Labnet) incubator bench top shaker at 37 °C and 1000 RPM. 10 μl of the overnight culture were added to 290 μl of LB, with antibiotics and 1 ng ml^−1^ of anhydrotetracycline (aTc) inducer or 50 mM of arabinose when required. For Supplementary Figure S2, acyl-homoserine lactone (AHL) [*N*-(β-ketocaproyl)-l-homoserine lactone, Cayman chemical] solution was also added with a final concentration of 10 and 100 μM as indicated. After 8–10 h, 50 μl of culture were transferred to a 96-well plate containing 50 μl of phosphate buffered saline (PBS) solution. An Infinite m1000 Pro plate reader (Tecan) was used to measure optical density (OD) at 600 nm, and to measure RFP fluorescence (FL) (540 nm excitation and 600 nm emission). For time course experiments shown in Supplementary Figure S5, cells were diluted 1:30 in LB and 100 μl were added to a 96-well plate, which was transferred to the plate reader with constant shaking, and OD and RFP measurements were taken every 10 min for 10 h.

### Bulk fluorescence data analysis

For each experiment there were two sets of controls: a media blank and *E. coli* MG1655 cells transformed with a combination of the control plasmids pJEC101, pJEC102, pJEC103 and pJEC598 (blank cells), which only contained the antibiotic resistance cassette and thus not expressing the reporter gene (Supplementary Figure S1). Each experiment contained four biological replicates of each control. OD and FL values for each colony were first corrected by subtracting the corresponding mean values of the media blank. The ratio of FL to OD (FL/OD) was then calculated for each well (grown from a single colony) and Normalized FL/OD values were obtained subtracting FL/OD of blank cells from the four colonies characterized. Means of FL/OD were calculated over replicates and error bars represent standard deviations (s.d.). To calculate fold activation, a no-sgRNA control was used in which cells were transformed with a control plasmid (pJEC102). Mean of FL/OD of the no-sgRNA control was calculated and used as a measurement of the basal expression state. Fold activation was calculated from this by dividing the FL/OD of the targeting sgRNA (experimental condition) by the no-sgRNA control. Error propagation was performed to obtain the s.d. values.

### Measuring distance-dependent effects

To evaluate distance-dependent effects, a library of seven sgRNAs (Supplementary Table S7) was programmed to target protospacer adjacent motifs (PAMs) located at positions 70, 80, 90 on the template strand and 61, 71, 81, 91 on the non-template strand upstream of the TSS of the RFP reporter plasmid (pJEC581). These same sgRNAs were used to target 9 additional reporter plasmids derived from pJEC581 (Reporter +1 to Reporter +9) that each contained additional nucleotides (1–9 nt) to extend the distance between the promoter TSS and each PAM (Supplementary Figure S6). As the basal RFP expression from each reporter varied slightly, the values are reported as fold activation, normalized against the no-sgRNA control for each reporter.

### Construction of phylogenetic tree

**Figure 2.**
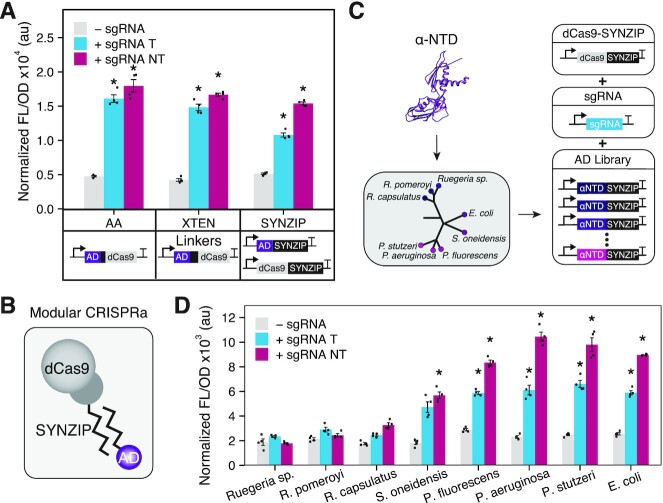
Evaluation of different ADs and fusion strategies. (**A**) Characterization of different protein fusion strategies to recruit the αNTD AD through dCas9 by a covalent two-alanine (AA) linker, covalent 16 amino acid XTEN linker, or a noncovalent SYNZIP interaction domain. Schematics of DNA constructs shown under each data set. (**B**) Schematic of the modular CRISPRa design. The dCas9 and AD are independently fused to SYNZIP domains that form heterodimers. (**C**) A schematic illustrating creation and screening of an AD library using our modular CRISPRa platform. Different ADs were identified from αNTDs derived from 8 different bacterial species and translationally fused to a SYNZIP domain. These AD-encoding plasmids were then co-transformed with dCas9-SYNZIP and sgRNA-encoding plasmids to create new CRISPRa systems. (**D**) Characterization of the AD library. Fluorescence characterization (measured in units of fluorescence [FL]/optical density [OD] at 600 nm) was performed with MG1655 *E. coli* cells transformed with an RFP reporter plasmid, a dCas9-AD plasmid or separate dCas9- and AD-encoding plasmids, and a sgRNA-encoding plasmid or a no-sgRNA control plasmid. sgRNA variants used targeted PAMs located at 80 bp upstream of the promoter TSS on the template strand (+ sgRNA T) and 81 bp upstream on the non-template stand (+ sgRNA NT). Data represent mean values and error bars represent s.d. of *n* = 4 biological replicates. A two tailed Student's t test was used to calculate p value comparing against the no-sgRNA control. * *P* < 0.0001; *P* > 0.0001 has no asterisk.

An expanded multifurcated phylogenetic tree, based on the NCBI taxonomy database, was generated using phyloT (https://phylot.biobyte.de/) to display the bacterial species from which αNTD sequences were obtained for data shown in Figure 2.

## RESULTS

### Identifying activation domains (ADs) for a bacterial CRISPRa system using dCas9 recruitment

As a starting point, our goal was to establish a basic CRISPRa system in *E. coli*. One of the most established routes to do this, is to fuse onto the N- or C-terminus of dCas9 the omega (ω) subunit of RNAP (28), which has served as a versatile AD in a variety of regulatory contexts (35,44) (Figure 1A). This is due to the ability of the ω subunit to structurally stabilize and recruit the β’ subunit of RNAP (45), functionalities that can activate transcription when localized close to promoter elements. To characterize this basic design, plasmids were constructed in which the ω subunit was fused to the C-terminus of the dCas9 using a two-alanine linker. To measure transcription activation, we constructed a reporter plasmid containing a constitutively expressed RFP gene with a J23117 promoter and multiple NGG protospacer adjacent motifs (PAMs) upstream of the promoter element, which was derived from a previously described plasmid (31,32) We designed corresponding sgRNA expression plasmids to target two PAMs located 80 bp upstream of the TSS in the template strand (80T) and 81 bp upstream of the TSS in the non-template strand (81NT). A plasmid containing only the antibiotic resistance cassette was constructed to be used as the no-sgRNA control. *E. coli* cells were transformed with the dCas9-ω plasmid, the RFP reporter plasmid, and either a sgRNA-encoding plasmid or the no-sgRNA control. Experiments were performed in two strains: *E. coli* K-12 BW25113 or an engineered *E. coli* K-12 BW25113 *ΔrpoZ* strain (43), in which the endogenous *rpoZ* gene encoding the ω subunit had been knocked out, which had previously shown to allow for greater activation from ω-based ADs (28,46). RFP fluorescence (540 nm excitation and 600 nm emission) and optical density at 600 nm were measured for each culture. From these experiments, we observed no significant activation when this CRISPRa system was targeted to either the template or non-template strand in the parental strain *E. coli* K-12 BW25113. However, in the knockout strain, targeting either DNA strand resulted in ∼8-fold activation (Figure 1B). These results confirmed that while the ω subunit of RNAP can serve as an AD, a significant limitation is its dependence on the use of a knockout strain. This is problematic because although the ω subunit is considered non-essential for many bacterial functions, its absence is known to reduce growth rate (45,47), inhibit antibiotic production (48), and inhibit morphological differentiation (49), potentially limiting the utility of these CRISPRa systems.

To optimize the activation of this CRISPRa system and remove the dependence on knockout strains, we next performed screening to identify alternative ADs. To do this, we identified a panel of ADs derived from the subunits of *E. coli* RNAP and bacterial transcription factors (Supplementary Table S3). Specifically, we evaluated the alpha subunit (α) and the α subunit N-terminal domain (αNTD). Our interest in the α subunit was due to previous demonstrations using it as an AD in two-hybrid screens (50), a function most likely explained by the role of the α subunit in initiating RNAP assembly (51). Additionally we used transcription factors from the AraC and LuxR family of regulators known to act as transcription activators through interactions with RNAP (52,53). For each AD, fusions to the N- and C-terminus of dCas9 were constructed, with the exception of SoxS for which only the C-terminal fusion could be obtained. These different fusions were then targeted to the RFP reporter plasmid at positions 80T and 81NT, and fluorescence was measured (Figure 1C). From these experiments, we observed that the αNTD fused to the N-terminus of the dCas9 was the strongest variant when targeted to both the non-template and template strand, achieving up to 5-fold activation. For experiments using LuxR as an AD, we also performed measurements in the presence of its inducer, *N*-acyl homoserine lactone (AHL) (Supplementary Figure S2), but no change in fluorescence was observed. In addition to this panel, we tested a recently described strong AD based on an evolved AsiA, an anti-sigma factor protein derived from T4 bacteriophage (33), targeting several upstream positions (Supplementary Figure S3). However, we did not observe significant activation, likely due to differences in our experimental system, including the use of different reporters and different sgRNA sequences. Taken together, these results demonstrate that the αNTD is an attractive AD for construction of a bacterial CRISPRa system.

### Exploring modular protein–protein interaction domains for AD recruitment

We next decided to investigate alternative protein fusion strategies to recruit the AD by dCas9. In particular, we were interested in utilizing noncovalent protein-protein interactions that would allow for the dCas9 and AD to be encoded on separate and easily interchangeable plasmids and thus allow for a high degree of modularity within our CRISPRa system. We envisaged this would be particularly valuable to eliminate the laborious cloning of the large dCas9-encoding plasmid and to allow for rapid screening of different synergistic combinations of the dCas9 and AD components. To this end, we decided to explore the use of synthetic coiled-coil SYNZIP interaction domains (39,40), which have provided a versatile protein interaction domain for molecular engineering applications. To test this, we constructed two new plasmids, one containing dCas9 fused to SYNZIP18 and the other containing the AD fused to SYNZIP17 (Supplementary Table S4), which form highly selective antiparallel heterodimers (Figure 2B, Supplementary Figure S4) (40,54). In order to benchmark the effectiveness of this approach, we compared it against two designs that used covalent linkers between the AD and the dCas9. These designs used linkers composed of either two alanines or a 16 amino acid sequence known as XTEN (SGSETPGTSESATPES), which is an artificial extended and unstructured linker that has seen broad utility for creating chimeric fusions with Cas proteins (55,56). Plasmids encoding these variants using the αNTD as the AD were constructed and characterized by targeting the RFP reporter plasmid at positions 80T and 81NT. This characterization revealed that in comparison to covalent linkers, the noncovalent linker maintained a similar level of activation fold, while offering increased modularity (Figure 2A). To demonstrate the inducibility of the modular CRISPRa system, we expressed SYNZIP-dCas9 under the control of a P_tet_ and P_bad_ promoters and the corresponding inducer was added. We observed that with P_tet,_ probably due to the leakiness of the promoter, more than 2-fold activation was obtained in the absence of the inducer and no further activation was achieved in the presence of the inducer. While P_bad_ promoter evidenced no activation in the absence of the inducer and >2-fold activation when the inducer was added. (Supplementary Figure S5A). We evaluated alternative designs of dCas9 fused to SYNZIP18, including fusions to the C- and N-terminus (dCas9-SYNZIP and SYNZIP-dCas9), and fusion of two tandem SYNZIP domains (dCas9–2x[SYNZIP]) (Supplementary Figure S5B). Similar levels of fold activation were achieved across these different design variants. In addition, we showed that growth (Supplementary Figure S5C) and FL levels (Supplementary Figure S5D) were not affected when the modular CRISPRa system was expressed without a sgRNA compared to cells expressing only the reporter.

To demonstrate the flexibility that our modular SYNZIP fusion approach allows, we screened a panel of seven additional ADs fused to SYNZIP17. As a proof of principle, we decided to screen a panel of αNTD derived from different species across the bacteria domain of life, to begin to understand the design rules governing this class of AD (Supplementary Table S6). Specifically, we obtained αNTD sequences from the genome of *Alphaproteobacteria*: *Ruegeria* sp. *TM 1040* (*Ruegeria* sp.), *Ruegeria pomeroyi* and *Rhodobacter capsulatus*, and *Gammaproteobacteria: Shewanella oneidensis*, *Pseudomonas fluorescens*, *Pseudomonas aeruginosa* and *Pseudomonas stutzeri* (Figure 2C). Characterizing these ADs in *E. coli* cells, we observed activation when targeting both the template and non-template strands with all of the αNTDs derived from *Gammaproteobacteria*, ranging from 2- to 5-fold activation (Figure 2D). Not surprisingly, the highest activation was obtained from *Pseudomonas* species, which are phylogenetically closer to *E. coli* and whose αNTD protein sequences share 69% sequence identity. In comparison, no activation was observed with the αNTD from *Ruegeria* species, which are more distantly related and share 44% protein sequence identity with *E. coli*. The successful activation using different αNTDs, despite different degrees of activation, suggests that αNTDs could be engineered to be used as an AD for CRISPRa systems across bacterial species.

### Distance-dependent activation patterns of our CRISPRa platform

**Figure 3.**
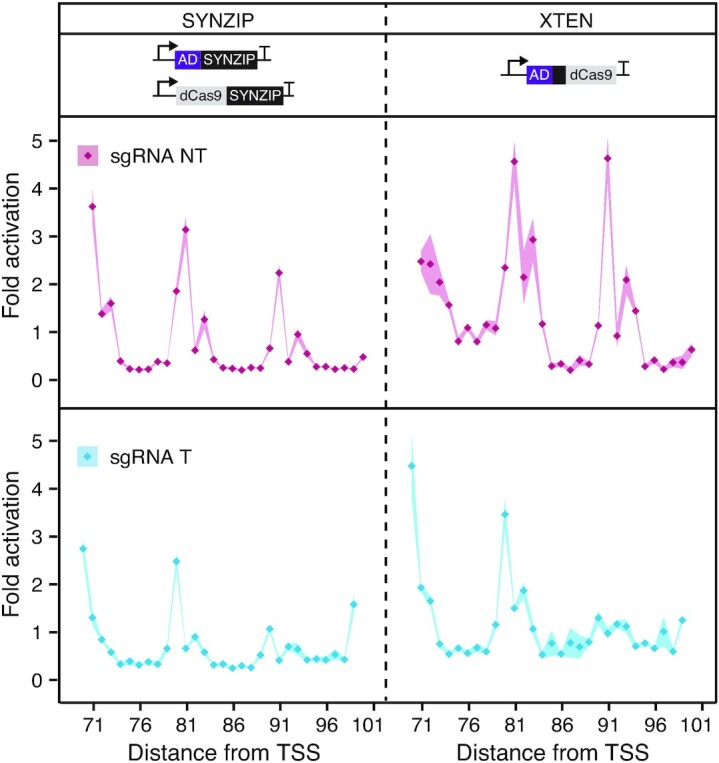
Characterization of distance-dependent activation patterns. Measuring the distance-dependent activation patterns of CRISPRa systems using XTEN and SYNZIP linkers. These systems were targeted to PAMs located between 71 and 101 bp upstream of the promoter TSS on both the template and non-template strands. Fluorescence characterization (measured in units of fluorescence [FL]/optical density [OD] at 600 nm) was performed in MG1655 *E. coli* cells transformed with an RFP reporter plasmid, a dCas9 plasmid, an AD plasmid and a sgRNA-encoding plasmid or a no-sgRNA control plasmid. Fold activation was calculated by dividing the [FL]/[OD] obtained in the presence of a targeting sgRNA against the no-sgRNA control within each reporter plasmid. Data represent mean values and shading represent s.d. of *n* = 4 biological replicates.

It has previously been shown that bacterial CRISPRa systems that utilize sgRNA-based recruitment of ADs (Supplementary Figure S4B) have periodical activation patterns in which activation is only observed when the system is targeted to a 2–4 bp window that repeats every ∼10–11 bp (32). Importantly, this means that for a given gene, only a fraction of target binding sites are functional, which significantly limits gene targeting range. For example, a previous analysis suggested only ∼10% of endogenous promoters in *E. coli* contained PAMs at the optimal targetable positions (32). To understand if these effects were observed for our modular CRISPRa design, we next characterized the distance-dependent activation pattern. To do this, we adapted a previously reported approach in which a panel of sgRNAs and base-shifted reporter plasmids are used to measure activation patterns at nucleotide resolution (Supplementary Figure 6, and Materials and Methods) (32). From these experiments, we observed periodic activation windows of 2–4 bp, which repeated every ∼10 bp (Figure 3). In order to understand if this was an inherent property of the SYNZIP design, or more generally, CRISPRa systems that recruit via dCas9, we also performed these experiments on a CRISPRa system that used the covalent XTEN linker (Figure 3). Interestingly, we observed that regardless of length or even type of linker used (i.e. covalent versus noncovalent linkers) activation patterns are conserved, with the highest activation being obtained in the same positions. These results showed for the first time that in addition to CRISPRa systems that utilize sgRNA-based recruitment of ADs, dCas9-based recruitment has similar distance-dependent periodic activation patterns. While the exact cause for this is unknown, we hypothesized that this was a result of the spatial localization of the AD by dCas9 relative to the bound DNA (32), which may only result in successful positioning of the AD to the nearby promoter when targeted on specific surfaces of the DNA double helix, which rotates every ∼10.5 bp.

### Expanding activation patterns using circularly permuted dCas9 variants

**Figure 4.**
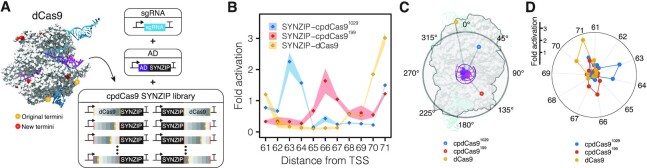
Circularly permuted dCas9 (cpdCas9) variants allow for an expanded target range. (**A**) cpdCas9 variants were fused to a SYNZIP through the new N- or C-termini to generate a cpdCas9 SYNZIP library that was evaluated as part of the modular CRISPRa system. cpdCas9 variants used in this study are shown on the crystal structure of Cas9 (PDB: 5F9R), DNA is colored purple, sgRNA is colored blue and dCas9 is colored grey. (**B**) Characterization of the distance-dependent activation patterns of CRISPRa systems using three dCas9 variants: wild-type dCas9 (SYNZIP-dCas9), cpdCas9^1029^ (SYNZIP-cpdCas9^1029^) and cpdCas9^199^ (SYNZIP-cpdCas9^199^). Distance-dependent activation patterns were measured by targeting PAMs located between 61 and 71 bp upstream of the promoter TSS on the non-template strand (61NT to 71NT). Fluorescence characterization (measured in units of fluorescence [FL]/optical density [OD] at 600 nm) was performed in MG1655 *E. coli* cells transformed with an RFP reporter plasmid, a plasmid encoding the cpdCas9 or dCas9 variant, a plasmid encoding αNTD-SYNZIP, and a sgRNA-encoding plasmid or a no-sgRNA control plasmid. Fold activation was calculated by dividing the [FL]/[OD] obtained in the presence of a targeting sgRNA against the no-sgRNA control within each reporter plasmid. Data represent mean values and shading represent s.d. of *n* = 4 biological replicates. (**C**) Structural analysis of the angles corresponding to the different positions of the N-terminal SYNZIP fusions in the dCas9, cpdCas9^1029,^and cpdCas9^199^ relative to the axis of the DNA double helix (purple). (**D**) Fold activation values obtained from the different dCas9 variants targeting positions 61NT to 71NT shown as a function of the targeted position in a DNA helical wheel that was set to 10.5 bp per turn.

In order to address this limitation, we decided to explore alternative dCas9 variants that could uniquely position ADs relative to the bound DNA, which we reasoned would create shifted activation patterns. To do this, we decided to use circularly permuted variants of dCas9 (cpdCas9) in which the original N- and C-termini were fused with a 20 amino acid linker and the order of the amino acid sequence changed to create new N- and C-termini (57). Our goal was to identify a library of different cpdCas9 variants that could be easily interchanged with the AD expression plasmids to create new CRISPRa systems capable of activating from distinct positions (Figure 4A). To initially test this idea, we performed a preliminary evaluation with a dCas9 variant circularly permuted at the 1029th residue (cpdCas9^1029^). A plasmid encoding an N-terminal SYNZIP fusion was created and combined with a sgRNA plasmid library to target this CRISPRa system to different PAMs located upstream of the RFP reporter gene on the non-template strand (Supplementary Figure S6 and S7). Comparison of the activation pattern of this CRISPRa system, compared to those obtained with wild-type dCas9, showed a ∼2 bp shifted activation pattern, resulting in activation from positions non-targetable by the wild-type dCas9 system (Figure 4B). Following this initial success, we decided to perform a more extensive screen to identify other potentially useful cpdCas9 variants. In particular, we wanted to identify variants able to activate from positions that appeared to be non-targetable by wild-type dCas9 or cpdCas9^1029^. To do this, we created N- and C-terminal fusions of the SYNZIP18 to 11 previously identified cpdCas9 variants (Supplementary Figure S8) and characterized their ability to activate from four PAMs located 65, 66, 67 and 68 bp upstream of the TSS in the non-template strand (Supplementary Figure S9). From this, we identified cpdCas9^199^ as a potential candidate, which was then characterized for its distance-dependent activation pattern (Figure 4B). From this, we confirmed that like cpdCas9^1029^, a CRISPRa system that utilized cpdCas9^199^ resulted in a shifted activation pattern. Interestingly, structural analysis of the new N-termini, to which the SYNZIP was fused in the dCas9, and their angles relative to the axis of the DNA double helix (Figure 4C) showed remarkable similarities to the relative angles of binding sites on the DNA double helix from which activation was observed (Figure 4D). This suggests that the shifted activation pattern could be explained as a result of distinct localization of ADs relative to the DNA double helix.

## DISCUSSION

In our work, we apply rational protein engineering to successfully generate and characterize a novel bacterial CRISPRa system presenting a high degree of modularity. Our system encodes the dCas9 and AD as plasmid-independent elements that can be easily exchanged, allowing each to be independently optimized and synergistically combined to create new CRISPRa systems with distinct properties. Through exchange of different ADs, we demonstrate that we can alter regulatory properties, such as fold activation, while exchanging dCas9 variants allows for distinct targeting range. As such, we believe the work described here enhances the current versatility of bacterial CRISPRa systems by creating a highly modular system with expanded target range.

Our screening of different ADs identified the N-terminal domain of the α subunit of RNAP (αNTD) to be a robust AD, able to successfully activate transcription when connected to dCas9 through either covalent or noncovalent linker domains. The α subunit of RNAP is naturally composed of two independently folded domains: αNTD and αCTD. In the process of RNAP assembly, αNTD dimerizes with a separate αNTD to initiate assembly, while αCTD is not required for this process, but instead interacts with transcription factors to recruit the RNAP to specific promoter sequences. Interestingly, we observed that the αNTD appeared to be optimal when fused through its C-terminus, in effect, mimicking its natural configuration in which the αCTD interacts with transcription factors to recruit RNAP. In this case, αCTD was replaced by dCas9 which performs the role of recruitment to a target promoter sequence. This activation by recruitment is analogous to regulatory mechanisms found in natural bacterial transcription activators, which rely on the recruitment of RNAP by protein-protein interactions. Interestingly, upon screening a library of αNTDs derived from different bacterial species, we observed a surprising level of portability. This is likely due to the high degree of sequence conservation across different species (58,59) and confirms previous observations that α subunits from different species can be functionally interchangeable (60,61). Thus, αNTD potentially provides an attractive AD as researchers attempt to transfer CRISPRa systems into non-model bacterial species. Given that αNTD activates via recruitment of RNAP, there is also potential to synergistically combine it with other classes of ADs that activate transcription through distinct mechanisms, for example, rearrangement of the promoter DNA structure (62).

The presence of distance-dependent periodic activation patterns has been previously reported for bacterial CRISPRa systems that utilize AD recruitment through interactions with the sgRNA (32). Here, we show that the periodic activation pattern is also present for our CRISPRa system that utilizes dCas9-based recruitment of ADs, and that this activation pattern is independent of the length or type (i.e. covalent or noncovalent) of linker connecting the AD to the dCas9. Interestingly, the activation pattern we obtained overlaps with the pattern previously reported, which also reported an inability to alter activation patterns through linker engineering (32). Based upon our analysis, it appears that this pattern is a result of spatial localization of the AD by dCas9, which appears to only result in activation when positioned on certain sides of the DNA double helix. This would also explain why the same activation patterns are observed when ADs are recruited through the sgRNA (32) or the N- and C-termini of dCas9, which are all located in close proximity within the tertiary structure of the dCas9-sgRNA complex (63). Exploiting this, we show that circularly permuted variants of dCas9 (cpdCas9) that have been engineered to contain new N- and C-termini at distinct positions within the tertiary structure, can be used to shift activation patterns and allow activation from distinct binding sites compared to wild-type dCas9. This significantly expands the number of binding sites from which CRISPRa systems can achieve activation, which we anticipate can be increased further in the future through more extensive screening of cpdCas9 variants. While in this study we demonstrated expanded targeting range using a synthetic σ^70^ promoter, we anticipate that this approach will allow activation of the large number of endogenous promoters containing PAM binding sites that are non-optimal for other CRISPRa systems (31–34). This approach is also complementary to other efforts to expand the target range of CRISPRa systems using PAM-relaxed dCas9 variants that can utilize alternative PAM sequences (32,64–66). We thus expect that combining cpdCas9 and PAM-relaxed dCas9 variants would provide even greater targeting range, as recently demonstrated for CRISPR–Cas base editor systems (67). Finally, harnessing CRISPR–Cas systems from different types and origins (68–70) could yield further systems with distinct regulatory properties.

This work, along with other recent efforts, have begun to address limitations of bacterial CRISPRa systems; however, challenges remain. First, in general bacterial CRISPRa systems have shown lower fold activation compared to their eukaryotic counterparts and other synthetic RNA-based activating systems (71–73), although recent efforts have identified promising ADs that can allow for high-fold activation (31–33). While low-fold activation is not necessarily a limitation for all applications, we anticipate that it will limit certain applications, such as the construction of complex synthetic gene circuits that depend on high dynamic ranges. Second, a deeper understanding of the rules governing activation patterns is required, particularly for endogenous promoter elements. Such design rules are critical to allow for reliable and even automated design of CRISPRa variants that are required to apply these technologies for large-scale genome-wide perturbation studies. Finally, more work is required to identify optimal ADs and understand the design rules of CRISPRa within non-model bacterial species. Given the remarkable progress made in recent years, we anticipate that these challenges and knowledge gaps will be addressed.

In summary, this work expands the current utility of bacterial CRISPRa systems by enhancing modularity and increasing targeting range. This work creates a novel platform for gene activation, which we anticipate will be of value for applications such as metabolic engineering, and to advance fundamental understanding of gene function and connectivity.

## DATA AVAILABILITY

All source data for main and SI figures was deposited in Rice University's Rice Digital Scholarship Archive (https://doi.org/10.25611/DG85-7Y09).

## Supplementary Material

gkab211_Supplemental_File
