# Supplementary material for: Rational engineering of a modular bacterial CRISPR–Cas activation platform with expanded target range
Source: Nucleic Acids Res. 2021 Apr 6;49(8):4793–802. doi: 10.1093/nar/gkab211 (PMC8096225; doi:10.1093/nar/gkab211)
Supplement: gkab211_Supplemental_File [file gkab211_supplemental_file.pdf]

Rational engineering of a modular bacterial CRISPR-Cas activation platform with expanded target range

Maria Claudia Villegas Kcam[1], Annette J. Tsong[1] and James Chappell[1,2]

1 - Department of BioSciences, Rice University, 6100 Main Street, MS 140, Houston, TX 77005, USA.
2 - Department of Bioengineering, Rice University, 6100 Main Street, MS 142, Houston, TX 77005, USA.
* Correspondance: Dr. James Chappell, email: jc125@rice.edu

TABLE OF CONTENTS:

**Page 2-6**	Supplementary Table 1. List of all plasmids used in this study
**Page 6-18**	Supplementary Table 2. Example of DNA plasmid sequences
**Page 18- 20**	Supplementary Table 3. Activator domain (AD) sequences used in this study
**Page 20**	Supplementary Table 4. SYNZIP sequences used in this study
**Page 21**	Supplementary Table 5. Covalent linker sequences used in this study
**Page 21-25**	Supplementary Table 6. Bacterial αNTD sequences
**Page 25**	Supplementary Table 7. sgRNA sequences used in this study
**Page 26**	Supplementary Figure 1. Maps of DNA plasmids used in this study
**Page 27**	Supplementary Figure 2. Evaluation of activation with a LuxR AD in the presence of N-acyl homoserine lactone (AHL)
**Page 28**	Supplementary Figure 3. Evaluation of AsiAm2.1 as an AD
**Page 29**	Supplementary Figure 4. Schematic of bacterial CRISPRa systems with different AD recruitment strategies
**Page 30**	Supplementary Figure 5. Characterization of SYNZIP fusions to dCas9
**Page 31**	Supplementary Figure 6. Reporter plasmids used to characterize activation patterns at nucleotide resolution
**Page 32**	Supplementary Figure 7. Characterization of activation with SYNZIP-cpdCas9^{1029}
**Page 33**	Supplementary Figure 8. Screening strategy to identify activating cpdCas9 variants
**Page 34**	Supplementary Figure 9. Screening of activation with the cpdCas9 library targeting the non-template strand
**Page 35**	References

Supplementary Table 1. List of all plasmids used in this study. Abbreviations are as follows: CamR = chloramphenicol resistance cassettes, AmpR = Ampicillin resistance cassettes, SpecR = Spectinomycin resistance, KanR = Kanamycin resistance, RFP = Red fluorescent protein, sgRNA = single guide RNA. Ribosomal binding sites (RBS): B0015, B0034, B0032, and BBa_J34801. Promoters: J23119, J23150, J23117, J23106, P_{tet}, P_{bad}. The constitutive promoters were obtained from the iGEM Registry of Standard Biological Parts (parts.igem.org). Origin of replication: p15A, ColE1, CloDF, and pSC101.

Plasmid ID	Plasmid architecture	Name	Figure
pJEC101	TrrnB – CamR – p15A origin	No dCas9	1,2,3,4, SI_2, SI_3, SI_5, SI_7, SI_8, SI_9
pJEC102	J23119 – TrrnB – ColE1 origin – AmpR	No-sgRNA control	1,2,3,4, SI_2, SI_3, SI_5, SI_7, SI_8, SI_9
pJEC103	J23119 – TrrnB – SpecR – CloDF origin	SpecR control	2,3,4, SI_5, SI_7, SI_8, Si_9
pJEC598	KanR – pSC101 origin	No reporter control	1,2,3,4, SI_2, SI_3, SI_5, SI_7, SI_8, SI_9
pJEC116	TetR – P_{tet} – B0034 – dCas9 – B0015– p15A origin – CamR	dCas9	SI_3, SI_5
pJEC547	TetR – P_{tet} – B0034 – dCas9 – 2alanine – ω – B0015– p15A origin – CamR	dCas9-ω	1
pJEC548	TetR – P_{tet} – B0034 – α – 2alanine – dCas9 – B0015– p15A origin– CamR	ω-dCas9	1
pJEC549	TetR – P_{tet} – B0034 – dCas9 – 2alanine – α– B0015– p15A origin– CamR	dCas9-α	1
pJEC550	TetR – P_{tet} – B0034–α– 2alanine – dCas9 – B0015– p15A origin– CamR	α-dCas9	1
pJEC551	TetR – P_{tet} – B0034 – dCas9 – 2alanine – αNTD – B0015– p15A origin– CamR	dCas9-αNTD	1
pJEC552	TetR – P_{tet} – B0034– αNTD – 2alanine – dCas9 – B0015– p15A origin– CamR	αNTD-dCas9	1,2
pJEC644	TetR – P_{tet} – B0034– dCas9 – 2alanine – SoxS – B0015– p15A origin– CamR	dCas9-SoxS	1
pJEC645	TetR – P_{tet} – B0034 – dCas9 – 2alanine – LuxR – B0015– p15A origin– CamR	dCas9-LuxR	1, SI_2
pJEC646	TetR – P_{tet} – B0034 – LuxR– dCas9 – 2alanine – B0015– p15A origin– CamR	LuxR-dCas9	1, SI_2

pJEC629	TetR – P_{tet} – B0034– αNTD – XTEN – dCas9 – B0015– p15A origin– CamR	αNTD-XTEN-dCas9	2, 3
pJEC556	TetR – P_{tet} – B0034 – SYNZIP18 – linker – dCas9 – B0015 – p15A origin– CamR	aTc inducible SYNZIP-dCas9	2, SI_5
pJEC585	J23150 – B0034– dCas9 – linker – SYNZIP18 – B0015 – p15A origin– CamR	dCas9-SYNZIP	2, SI_5
pJEC578	J23150 – B0034 – SYNZIP18 – linker – dCas9 – B0015 – p15A origin– CamR	SYNZIP-dCas9	3,4, SI_5, SI_7
pJEC570	J23150 – B0034– dCas9 – linker – SYNZIP18 – SYNZIP18 – B0015 – p15A origin– CamR	dCas9-2X(SYNZIP)	SI_5
pJEC691	AraC – P_{bad} – B0034 – SYNZIP18 – linker – dCas9 – B0015 – p15A origin– CamR	Arabinose inducible SYNZIP-dCas9	SI_5
pJEC581	KanR – PAM rich sequence – J23117 – BBa_J34801 – mRFP – TrrnB – pSC101 origin	Reporter	1,2,3,4, SI_2, SI_3, SI_5, SI_7, SI_8
pJEC597	KanR – PAM rich sequence +1 – J23117 – BBa_J34801 – mRFP – TrrnB – pSC101 origin	Reporter+1	3,4, SI_3, SI_7, SI_8
pJEC596	KanR – PAM rich sequence +2 – J23117 – BBa_J34801 – mRFP – TrrnB – pSC101 origin	Reporter+2	3,4, SI_7, SI_8
pJEC595	KanR – PAM rich sequence +3 – J23117 – BBa_J34801 – mRFP – TrrnB – pSC101 origin	Reporter+3	3,4, SI_7, SI_8
pJEC594	KanR – PAM rich sequence +4 – J23117 – BBa_J34801 – mRFP – TrrnB – pSC101 origin	Reporter+4	3,4, SI_3, SI_7, SI_8, SI_9
pJEC593	KanR – PAM rich sequence +5 – J23117 – BBa_J34801 – mRFP – TrrnB – pSC101 origin	Reporter+5	3,4, SI_7, SI_8, SI_9
pJEC592	KanR – PAM rich sequence +6 – J23117 – BBa_J34801 – mRFP – TrrnB – pSC101 origin	Reporter+6	3,4, SI_7, SI_9, SI_9
pJEC591	KanR – PAM rich sequence +7 – J23117 – BBa_J34801 – mRFP – TrrnB – pSC101 origin	Reporter+7	3,4, SI_3, SI_7, SI_8, SI_9
pJEC590	KanR – PAM rich sequence +8 – J23117 – BBa_J34801 – mRFP – TrrnB – pSC101 origin	Reporter+8	3,4, SI_7, SI_8
pJEC589	KanR – PAM rich sequence +9 – J23117 – BBa_J34801 – mRFP – TrrnB – pSC101 origin	Reporter+9	3,4, SI_7, SI_8

pJEC626	J23106 – B0032 – αNTD – linker – SYNZIP17 – TrrnB – SpecR – CloDF origin	Activator-SYNZIP	2,3,4, SI_5, SI_7, SI_8, SI_9
pJEC608	J23119 – sgRNA70T – TrrnB – ColE1 origin – AmpR	sgRNA 70T	3
pJEC567	J23119 – sgRNA80T – TrrnB – ColE1 origin – AmpR	sgRNA 80T	1, 2, 3
pJEC609	J23119 – sgRNA90T – TrrnB – ColE1 origin – AmpR	sgRNA 90T	3, SI_3
pJEC583	J23119 – sgRNA100T – TrrnB – ColE1 origin – AmpR	sgRNA 100T	SI_3
pJEC564	J23119 – sgRNA61NT – TrrnB – ColE1 origin – AmpR	sgRNA 61NT	4, SI_7, SI_8, SI_9
pJEC610	J23119 – sgRNA71NT – TrrnB – ColE1 origin – AmpR	sgRNA 71NT	3, SI_7
pJEC566	J23119 – sgRNA81NT – TrrnB – ColE1 origin – AmpR	sgRNA 81NT	1, 2, 3, SI_2, SI_3, SI_5
pJEC611	J23119 – sgRNA91NT – TrrnB – ColE1 origin – AmpR	sgRNA 91NT	3
pJEC584	J23119 – sgRNA121NT – TrrnB – ColE1 origin – AmpR	sgRNA 121NT	SI_3
pJEC605	J23150 – B0034 – SYNZIP18 – linker – cpdCas9^{1029}– B0015 – p15A origin– CamR	SYNZIP-cpdCas9^{1029}	4, SI_7
pJEC647	J23150 – B0034– cpdCas9^{199}– linker – SYNZIP18 – B0015 – p15A origin– CamR	cpdCas9^{199}-SYNZIP	SI_9
pJEC648	J23150 – B0034– cpdCas9^{181}– linker – SYNZIP18 – B0015 – p15A origin– CamR	cpdCas9^{181}-SYNZIP	SI_9
pJEC649	J23150 – B0034– cpdCas9^{230}– linker – SYNZIP18 – B0015 – p15A origin– CamR	cpdCas9^{230}-SYNZIP	SI_9
pJEC650	J23150 – B0034– cpdCas9^{270}– linker – SYNZIP18 – B0015 – p15A origin– CamR	cpdCas9^{270}-SYNZIP	SI_9
pJEC651	J23150 – B0034– cpdCas9^{310}– linker – SYNZIP18 – B0015 – p15A origin– CamR	cpdCas9^{310}-SYNZIP	SI_9
pJEC652	J23150 – B0034– cpdCas9^{1010}– linker – SYNZIP18 – B0015 – p15A origin– CamR	cpdCas9^{1010}-SYNZIP	SI_9
pJEC653	J23150 – B0034– cpdCas9^{1016}– linker – SYNZIP18 – B0015 – p15A origin– CamR	cpdCas9^{1016}-SYNZIP	SI_9
pJEC654	J23150 – B0034– cpdCas9^{1023}– linker – SYNZIP18 – B0015 – p15A origin– CamR	cpdCas9^{1023}-SYNZIP	SI_9

pJEC655	J23150 – B0034– cpdCas9^{1041}– linker – SYNZIP18 – B0015 – p15A origin– CamR	cpdCas9^{1041}-SYNZIP	SI_9
pJEC656	J23150 – B0034– cpdCas9^{1249}– linker – SYNZIP18 – B0015 – p15A origin– CamR	cpdCas9^{1249}-SYNZIP	SI_9
pJEC657	J23150 – B0034– cpdCas9^{1282}– linker – SYNZIP18 – B0015 – p15A origin– CamR	cpdCas9^{1282}-SYNZIP	SI_9
pJEC658	J23150 – B0034 – SYNZIP18 – linker – cpdCas9^{199}– B0015 – p15A origin– CamR	SYNZIP-cpdCas9^{199}	4, SI_9
pJEC659	J23150 – B0034 – SYNZIP18 – linker – cpdCas9^{181}B0015 – p15A origin– CamR	SYNZIP-cpdCas9^{181}	SI_9
pJEC660	J23150 – B0034 – SYNZIP18 – linker – cpdCas9^{230}– B0015 – p15A origin– CamR	SYNZIP-cpdCas9^{230}	SI_9
pJEC661	J23150 – B0034 – SYNZIP18 – linker – cpdCas9^{270}– B0015 – p15A origin– CamR	SYNZIP-cpdCas9^{270}	SI_9
pJEC662	J23150 – B0034 – SYNZIP18 – linker – cpdCas9^{310}– B0015 – p15A origin– CamR	SYNZIP-cpdCas9^{310}	SI_9
pJEC663	J23150 – B0034 – SYNZIP18 – linker – cpdCas9^{1010}– B0015 – p15A origin– CamR	SYNZIP-cpdCas9^{1010}	SI_9
pJEC664	J23150 – B0034 – SYNZIP18 – linker – cpdCas9^{1016}– B0015 – p15A origin– CamR	SYNZIP-cpdCas9^{1016}	SI_9
pJEC665	J23150 – B0034 – SYNZIP18 – linker – cpdCas9^{1023}– B0015 – p15A origin– CamR	SYNZIP-cpdCas9^{1023}	SI_9
pJEC666	J23150 – B0034 – SYNZIP18 – linker – cpdCas9^{1041}– B0015 – p15A origin– CamR	SYNZIP-cpdCas9^{1041}	SI_9
pJEC667	J23150 – B0034 – SYNZIP18 – linker – cpdCas9^{1249}– B0015 – p15A origin– CamR	SYNZIP-cpdCas9^{1249}	SI_9
pJEC668	J23150 – B0034 – SYNZIP18 – linker – cpdCas9^{1282}– B0015 – p15A origin– CamR	SYNZIP-cpdCas9^{1282}	SI_9
pJEC637	J23106 – B0032 – αNTD$^{P.\ aeruginosa}$ – linker – SYNZIP17 – TrrnB – SpecR – CloDF origin	P. aeruginosa αNTD	2
pJEC638	J23106 – B0032 – αNTD$^{P.\ fluorescens}$ – linker – SYNZIP17 – TrrnB – SpecR – CloDF origin	P. fluorescens αNTD	2

pJEC639	J23106 – B0032 – αNTD$^{P. stutzeri}$ – linker – SYNZIP17 – TrrnB – SpecR – CloDF origin	P. stutzeri αNTD	2
pJEC640	J23106 – B0032 – αNTD$^{R. pomeroyi}$ – linker – SYNZIP17 – TrrnB – SpecR – CloDF origin	R. pomeroyi αNTD	2
pJEC641	J23106 – B0032 – αNTD$^{Ruegeria\ sp.}$ – linker – SYNZIP17 – TrrnB – SpecR – CloDF origin	Ruegeria sp. αNTD	2
pJEC642	J23106 – B0032 – αNTD$^{R. capsulatus}$ – linker – SYNZIP17 – TrrnB – SpecR – CloDF origin	R. capsulatus αNTD	2
pJEC643	J23106 – B0032 – αNTD$^{S. oneidensis}$ – linker – SYNZIP17 – TrrnB – SpecR – CloDF origin	S. oneidensis αNTD	2
addgene ID #158065	TetR – P_{tet} – B0034 – dCas9_AsiA_m2.1 – B0015– p15A origin – CamR	pdCas9_AsiA_m2.1	SI_3

Supplementary Table 2. Example of DNA plasmid sequences.

Name	DNA sequence
Example of dCas9 plasmid with C-terminal fusion pJEC547 (Promoter-RBS-dCas9-linker-activator-Terminator-p15A-Cam)	GACGTCTTAAGACCCACTTTCACATTTAAGTTGTTTTTCTAATCCGCATAT<br>GATCAATTCAAGGCCGAATAAGAAGGCTGGCTCTGCACCTTGGTGATCAA<br>ATAATTCGATAGCTTGTCGTAATAATGGCGGCATACTATCAGTAGTAGGTG<br>TTTCCCTTTCTTCTTTAGCGACTTGATGCTCTTGATCTTCCAATACGCAAC<br>CTAAAGTAAAATGCCCCACAGCGCTGAGTGCATATAATGCATTCTCTAGT<br>GAAAAACCTTGTTGGCATAAAAAGGCTAATTGATTTTCGAGAGTTTCATAC<br>TGTTTTTCTGTAGGCCGTGTACCTAAATGTACTTTTGCTCCATCGCGATGA<br>CTTAGTAAAGCACATCTAAAACTTTTAGCGTTATTACGTAAAAAATCTTGCC<br>AGCTTTCCCCTTCTAAAGGGCAAAAGTGAGTATGGTGCCTATCTAACATCT<br>CAATGGCTAAGGCGTCGAGCAAAGCCCGCTTATTTTTTACATGCCAATAC<br>AATGTAGGCTGCTCTACACCTAGCTTCTGGGCGAGTTTACGGGTTGTTAA<br>ACCTTCGATTCCGACCTCATTAAGCAGCTCTAATGCGCTGTTAATCACTTT<br>ACTTTTATCTAATCTAGACATCATTAATTCCTAATTTTTGTTGACACTCTATC<br>GTTGATAGAGTTATTTTACCACTCCCTATCAGTGATAGAGAAAAGAATTCA<br>AAAGATCTAAAGAGGAGAAAGGATCTATGGATAAGAAATACTCAATAGGC<br>TTAGCTATCGGCACAAATAGCGTCGGATGGGCGGTGATCACTGATGAATA<br>TAAGGTTCCGTCTAAAAAGTTCAAGGTTCTGGGAAATACAGACCGCCACA<br>GTATCAAAAAAAATCTTATAGGGGCTCTTTTATTTGACAGTGGAGAGACAG<br>CGGAAGCGACTCGTCTCAAACGGACAGCTCGTAGAAGGTATACACGTCG<br>GAAGAATCGTATTTGTTATCTACAGGAGATTTTTTCAAATGAGATGGCGAA<br>AGTAGATGATAGTTTCTTTCATCGACTTGAAGAGTCTTTTTTGGTGGAAGA<br>AGACAAGAAGCATGAACGTCATCCTATTTTTGGAAATATAGTAGATGAAGT<br>TGCTTATCATGAGAAATATCCAACTATCTATCATCTGCGAAAAAAATTGGT<br>AGATTCTACTGATAAAGCGGATTTGCGCTTAATCTATTTGGCCTTAGCGCA<br>TATGATTAAGTTTCGTGGTCATTTTTTGATTGAGGGAGATTTAAATCCTGAT<br>AATAGTGATGTGGACAAACTATTTATCCAGTTGGTACAAACCTACAATCAA<br>TTATTTGAAGAAAACCCTATTAACGCAAGTGGAGTAGATGCTAAAGCGATT

	CTTTCTGCACGATTGAGTAAATCAAGACGATTAGAAAATCTCATTGCTCAG<br>CTCCCCGGTGAGAAGAAAAATGGCTTATTTGGGAATCTCATTGCTTTGTC<br>ATTGGGTTTGACCCCTAATTTTAAATCAAATTTTGATTTGGCAGAAGATGC<br>TAAATTACAGCTTTCAAAAGATACTTACGATGATGATTTAGATAATTTATTG<br>GCGCAAATTGGAGATCAATATGCTGATTTGTTTTTGGCAGCTAAGAATTTA<br>TCAGATGCTATTTTACTTTCAGATATCCTAAGAGTAAATACTGAAATAACTA<br>AGGCTCCCCTATCAGCTTCAATGATTAAACGCTACGATGAACATCATCAA<br>GACTTGACTCTTTTAAAAGCTTTAGTTCGACAACAACTTCCAGAAAAGTAT<br>AAAGAAATCTTTTTTGATCAATCAAAAAACGGATATGCAGGTTATATTGAT<br>GGGGGAGCTAGCCAAGAAGAATTTTATAAATTTATCAAACCAATTTTAGAA<br>AAAATGGATGGTACTGAGGAATTATTGGTGAAACTAAATCGTGAAGATTTG<br>CTGCGCAAGCAACGGACCTTTGACAACGGCTCTATTCCCCATCAAATTCA<br>CTTGGGTGAGCTGCATGCTATTTTGAGAAGACAAGAAGACTTTTATCCATT<br>TTTAAAAGACAATCGTGAGAAGATTGAAAAAATCTTGACTTTTCGAATTCC<br>TTATTATGTTGGTCCATTGGCGCGTGGCAATAGTCGTTTTGCATGGATGA<br>CTCGGAAGTCTGAAGAAACAATTACCCCATGGAATTTTGAAGAAGTTGTC<br>GATAAAGGTGCTTCAGCTCAATCATTTATTGAACGCATGACAAACTTTGAT<br>AAAAATCTTCCAAATGAAAAAGTACTACCAAAACATAGTTTGCTTTATGAGT<br>ATTTTACGGTTTATAACGAATTGACAAAGGTCAAATATGTTACTGAAGGAA<br>TGCGAAAACCAGCATTTCTTTCAGGTGAACAGAAGAAAGCCATTGTTGATT<br>TACTCTTCAAAACAAATCGAAAAGTAACCGTTAAGCAATTAAAAGAAGATT<br>ATTTCAAAAAAATAGAATGTTTTGATAGTGTTGAAATTTCAGGAGTTGAAG<br>ATAGATTTAATGCTTCATTAGGTACCTACCATGATTTGCTAAAAATTATTAA<br>AGATAAAGATTTTTTGGATAATGAAGAAAATGAAGATATCTTAGAGGATAT<br>TGTTTTAACATTGACCTTATTTGAAGATAGGGAGATGATTGAGGAAAGACT<br>TAAAACATATGCTCACCTCTTTGATGATAAGGTGATGAAACAGCTTAAACG<br>TCGCCGTTATACTGGTTGGGGACGTTTGTCTCGAAAATTGATTAATGGTAT<br>TAGGGATAAGCAATCTGGCAAAACAATATTAGATTTTTTGAAATCAGATGG<br>TTTTGCCAATCGCAATTTTATGCAGCTGATCCATGATGATAGTTTGACATT<br>TAAAGAAGACATTCAAAAAGCACAAGTGTCTGGACAAGGCGATAGTTTAC<br>ATGAACATATTGCAAATTTAGCTGGTAGCCCTGCTATTAAAAAAGGTATTT<br>TACAGACTGTAAAAGTTGTTGATGAATTGGTCAAAGTAATGGGGCGGCAT<br>AAGCCAGAAAATATCGTTATTGAAATGGCACGTGAAAATCAGACAACTCAA<br>AAGGGCCAGAAAAATTCGCGAGAGCGTATGAAACGAATCGAAGAAGGTA<br>TCAAAGAATTAGGAAGTCAGATTCTTAAAGAGCATCCTGTTGAAAATACTC<br>AATTGCAAAATGAAAAGCTCTATCTCTATTATCTCCAAAATGGAAGAGACA<br>TGTATGTGGACCAAGAATTAGATATTAATCGTTTAAGTGATTATGATGTCG<br>ATGCCATTGTTCCACAAAGTTTCCTTAAAGACGATTCAATAGACAATAAGG<br>TCTTAACGCGTTCTGATAAAAATCGTGGTAAATCGGATAACGTTCCAAGTG<br>AAGAAGTAGTCAAAAAGATGAAAAACTATTGGAGACAACTTCTAAACGCCA<br>AGTTAATCACTCAACGTAAGTTTGATAATTTAACGAAAGCTGAACGTGGAG<br>GTTTGAGTGAACTTGATAAAGCTGGTTTTATCAAACGCCAATTGGTTGAAA<br>CTCGCCAAATCACTAAGCATGTGGCACAAATTTTGGATAGTCGCATGAAT<br>ACTAAATACGATGAAAATGATAAACTTATTCGAGAGGTTAAAGTGATTACC<br>TTAAAATCTAAATTAGTTTCTGACTTCCGAAAAGATTTCCAATTCTATAAAG<br>TACGTGAGATTAACAATTACCATCATGCCCATGATGCGTATCTAAATGCCG<br>TCGTTGGAACTGCTTTGATTAAGAAATATCCAAAACTTGAATCGGAGTTTG<br>TCTATGGTGATTATAAAGTTTATGATGTTCGTAAAATGATTGCTAAGTCTGA<br>GCAAGAAATAGGCAAAGCAACCGCAAAATATTTCTTTTACTCTAATATCAT<br>GAACTTCTTCAAAACAGAAATTACACTTGCAAATGGAGAGATTCGCAAACG<br>CCCTCTAATCGAAACTAATGGGGAAACTGGAGAAATTGTCTGGGATAAAG

	GGCGAGATTTTGCCACAGTGCGCAAAGTATTGTCCATGCCCCAAGTCAAT<br>ATTGTCAAGAAAACAGAAGTACAGACAGGCGGATTCTCCAAGGAGTCAAT<br>TTTACCAAAAAGAAATTCGGACAAGCTTATTGCTCGTAAAAAAGACTGGGA<br>TCCAAAAAAATATGGTGGTTTTGATAGTCCAACGGTAGCTTATTCAGTCCT<br>AGTGGTTGCTAAGGTGGAAAAAGGGAAATCGAAGAAGTTAAAATCCGTTA<br>AAGAGTTACTAGGGATCACAATTATGGAAAGAAGTTCCTTTGAAAAAAATC<br>CGATTGACTTTTTAGAAGCTAAAGGATATAAGGAAGTTAAAAAAGACTTAA<br>TCATTAAACTACCTAAATATAGTCTTTTTGAGTTAGAAAACGGTCGTAAAC<br>GGATGCTGGCTAGTGCCGGAGAATTACAAAAAGGAAATGAGCTGGCTCT<br>GCCAAGCAAATATGTGAATTTTTTATATTTAGCTAGTCATTATGAAAAGTTG<br>AAGGGTAGTCCAGAAGATAACGAACAAAAACAATTGTTTGTGGAGCAGCA<br>TAAGCATTATTTAGATGAGATTATTGAGCAAATCAGTGAATTTTCTAAGCG<br>TGTTATTTTAGCAGATGCCAATTTAGATAAAGTTCTTAGTGCATATAACAAA<br>CATAGAGACAAACCAATACGTGAACAAGCAGAAAATATTATTCATTTATTT<br>ACGTTGACGAATCTTGGAGCTCCCGCTGCTTTTAAATATTTTGATACAACA<br>ATTGATCGTAAACGATATACGTCTACAAAAGAAGTTTTAGATGCCACTCTT<br>ATCCATCAATCCATCACTGGTCTTTATGAAACACGCATTGATTTGAGTCAG<br>CTAGGAGGTGACGCAGCTGCACGCGTAACTGTTCAGGACGCTGTAGAGA<br>AAATTGGTAACCGTTTTGACCTGGTACTGGTCGCCGCGCGTCGCGCTCG<br>TCAGATGCAGGTAGGCGGAAAGGATCCGCTGGTACCGGAAGAAAACGAT<br>AAAACCACTGTAATCGCGCTGCGCGAAATCGAAGAAGGTCTGATCAACAA<br>CCAGATCCTCGACGTTCGCGAACGCCAGGAACAGCAAGAGCAGGAAGCC<br>GCTGAATTACAAGCCGTTACCGCTATTGCTGAAGGTCGTCGTTAACTCGA<br>GTAAGGATCTCCAGGCATCAAATAAAACGAAAGGCTCAGTCGAAAGACTG<br>GGCCTTTCGTTTTATCTGTTGTTTGTCGGTGAACGCTCTCTACTAGAGTCA<br>CACTGGCTCACCTTCGGGTGGGCCTTTCTGCGTTTATACCTAGGGATATA<br>TTCCGCTTCCTCGCTCACTGACTCGCTACGCTCGGTCGTTCGACTGCGG<br>CGAGCGGAAATGGCTTACGAACGGGGCGGAGATTTCCTGGAAGATGCCA<br>GGAAGATACTTAACAGGGAAGTGAGAGGGCCGCGGCAAAGCCGTTTTTC<br>CATAGGCTCCGCCCCCCTGACAAGCATCACGAAATCTGACGCTCAAATCA<br>GTGGTGGCGAAACCCGACAGGACTATAAAGATACCAGGCGTTTCCCCCT<br>GGCGGCTCCCTCGTGCGCTCTCCTGTTCCTGCCTTTCGGTTTACCGGTGT<br>CATTCCGCTGTTATGGCCGCGTTTGTCTCATTCCACGCCTGACACTCAGT<br>TCCGGGTAGGCAGTTCGCTCCAAGCTGGACTGTATGCACGAACCCCCCG<br>TTCAGTCCGACCGCTGCGCCTTATCCGGTAACTATCGTCTTGAGTCCAAC<br>CCGGAAAGACATGCAAAAGCACCACTGGCAGCAGCCACTGGTAATTGATT<br>TAGAGGAGTTAGTCTTGAAGTCATGCGCCGGTTAAGGCTAAACTGAAAGG<br>ACAAGTTTTGGTGACTGCGCTCCTCCAAGCCAGTTACCTCGGTTCAAAGA<br>GTTGGTAGCTCAGAGAACCTTCGAAAAACCGCCCTGCAAGGCGGTTTTTT<br>CGTTTTCAGAGCAAGAGATTACGCGCAGACCAAAACGATCTCAAGAAGAT<br>CATCTTATTAATCAGATAAAATATTTCTAGATTTCAGTGCAATTTATCTCTT<br>CAAATGTAGCACCTGAAGTCAGCCCCATACGATATAAGTTGTTACTAGTG<br>CTTGGATTCTCACCAATAAAAAACGCCCGGCGGCAACCGAGCGTTCTGAA<br>CAAATCCAGATGGAGTTCTGAGGTCATTACTGGATCTATCAACAGGAGTC<br>CAAGCGAGCTCGATATCAAATTACGCCCCGCCCTGCCACTCATCGCAGTA<br>CTGTTGTAATTCATTAAGCATTCTGCCGACATGGAAGCCATCACAAACGG<br>CATGATGAACCTGAATCGCCAGCGGCATCAGCACCTTGTCGCCTTGCGTA<br>TAATATTTGCCCATGGTGAAAACGGGGGCGAAGAAGTTGTCCATATTGGC<br>CACGTTTAAATCAAAACTGGTGAAACTCACCCAGGGATTGGCTGAGACGA<br>AAAACATATTCTCAATAAACCCTTTAGGGAAATAGGCCAGGTTTTCACCGT<br>AACACGCCACATCTTGCGAATATATGTGTAGAAACTGCCGGAAATCGTCG

	TGGTATTCACTCCAGAGCGATGAAAACGTTTCAGTTTGCTCATGGAAAAC<br>GGTGTAACAAGGGTGAACACTATCCCATATCACCAGCTCACCGTCTTTCA<br>TTGCCATACGAAATTCCGGATGAGCATTCATCAGGCGGGCAAGAATGTGA<br>ATAAAGGCCGGATAAAACTTGTGCTTATTTTTCTTTACGGTCTTTAAAAAG<br>GCCGTAATATCCAGCTGAACGGTCTGGTTATAGGTACATTGAGCAACTGA<br>CTGAAATGCCTCAAAATGTTCTTTACGATGCCATTGGGATATATCAACGGT<br>GGTATATCCAGTGATTTTTTTCTCCATTTTAGCTTCCTTAGCTCCTGAAAAT<br>CTCGATAACTCAAAAAATACGCCCGGTAGTGATCTTATTTCATTATGGTGA<br>AAGTTGGAACCTCTTACGTGCCGATCAACGTCTCATTTTCGCCAGATATC
Example dCas9 plasmid with N-terminal fusion pJEC548 (Promoter-RBS – activator-linker-dCas9-Terminator-p15A-Cam)	GACGTCTTAAGACCCACTTTCACATTTAAGTTGTTTTTCTAATCCGCATAT<br>GATCAATTCAAGGCCGAATAAGAAGGCTGGCTCTGCACCTTGGTGATCAA<br>ATAATTCGATAGCTTGTCGTAATAATGGCGGCATACTATCAGTAGTAGGTG<br>TTTCCCTTTCTTCTTTAGCGACTTGATGCTCTTGATCTTCCAATACGCAAC<br>CTAAAGTAAAATGCCCCACAGCGCTGAGTGCATATAATGCATTCTCTAGT<br>GAAAAACCTTGTTGGCATAAAAAGGCTAATTGATTTTCGAGAGTTTCATAC<br>TGTTTTTCTGTAGGCCGTGTACCTAAATGTACTTTTGCTCCATCGCGATGA<br>CTTAGTAAAGCACATCTAAAACTTTTAGCGTTATTACGTAAAAAATCTTGCC<br>AGCTTTCCCCTTCTAAAGGGCAAAAGTGAGTATGGTGCCTATCTAACATCT<br>CAATGGCTAAGGCGTCGAGCAAAGCCCGCTTATTTTTTACATGCCAATAC<br>AATGTAGGCTGCTCTACACCTAGCTTCTGGGCGAGTTTACGGGTTGTTAA<br>ACCTTCGATTCCGACCTCATTAAGCAGCTCTAATGCGCTGTTAATCACTTT<br>ACTTTTATCTAATCTAGACATCATTAATTCCTAATTTTTGTTGACACTCTATC<br>GTTGATAGAGTTATTTTACCACTCCCTATCAGTGATAGAGAAAAGAATTCA<br>AAAGATCTAAAGAGGAGAAAGGATCTATGGCACGCGTAACTGTTCAGGAC<br>GCTGTAGAGAAAATTGGTAACCGTTTTGACCTGGTACTGGTCGCCGCGC<br>GTCGCGCTCGTCAGATGCAGGTAGGCGGAAAGGATCCGCTGGTACCGG<br>AAGAAAACGATAAAACCACTGTAATCGCGCTGCGCGAAATCGAAGAAGGT<br>CTGATCAACAACCAGATCCTCGACGTTCGCGAACGCCAGGAACAGCAAG<br>AGCAGGAAGCCGCTGAATTACAAGCCGTTACCGCTATTGCTGAAGGTCGT<br>CGTTAAGCTGCAGATAAGAAATACTCAATAGGCTTAGCTATCGGCACAAA<br>TAGCGTCGGATGGGCGGTGATCACTGATGAATATAAGGTTCCGTCTAAAA<br>AGTTCAAGGTTCTGGGAAATACAGACCGCCACAGTATCAAAAAAAATCTTA<br>TAGGGGCTCTTTTATTTGACAGTGGAGAGACAGCGGAAGCGACTCGTCTC<br>AAACGGACAGCTCGTAGAAGGTATACACGTCGGAAGAATCGTATTTGTTA<br>TCTACAGGAGATTTTTTCAAATGAGATGGCGAAAGTAGATGATAGTTTCTT<br>TCATCGACTTGAAGAGTCTTTTTTGGTGGAAGAAGACAAGAAGCATGAAC<br>GTCATCCTATTTTTGGAAATATAGTAGATGAAGTTGCTTATCATGAGAAAT<br>ATCCAACTATCTATCATCTGCGAAAAAAATTGGTAGATTCTACTGATAAAG<br>CGGATTTGCGCTTAATCTATTTGGCCTTAGCGCATATGATTAAGTTTCGTG<br>GTCATTTTTTGATTGAGGGAGATTTAAATCCTGATAATAGTGATGTGGACA<br>AACTATTTATCCAGTTGGTACAAACCTACAATCAATTATTTGAAGAAAACC<br>CTATTAACGCAAGTGGAGTAGATGCTAAAGCGATTCTTTCTGCACGATTG<br>AGTAAATCAAGACGATTAGAAAATCTCATTGCTCAGCTCCCCGGTGAGAA<br>GAAAAATGGCTTATTTGGGAATCTCATTGCTTTGTCATTGGGTTTGACCCC<br>TAATTTTAAATCAAATTTTGATTTGGCAGAAGATGCTAAATTACAGCTTTCA<br>AAAGATACTTACGATGATGATTTAGATAATTTATTGGCGCAAATTGGAGAT<br>CAATATGCTGATTTGTTTTTGGCAGCTAAGAATTTATCAGATGCTATTTTAC<br>TTTCAGATATCCTAAGAGTAAATACTGAAATAACTAAGGCTCCCCTATCAG<br>CTTCAATGATTAAACGCTACGATGAACATCATCAAGACTTGACTCTTTTAA<br>AAGCTTTAGTTCGACAACAACTTCCAGAAAAGTATAAAGAAATCTTTTTTG<br>ATCAATCAAAAAACGGATATGCAGGTTATATTGATGGGGGAGCTAGCCAA

	GAAGAATTTTATAAATTTATCAAACCAATTTTAGAAAAAATGGATGGTACTG<br>AGGAATTATTGGTGAAACTAAATCGTGAAGATTTGCTGCGCAAGCAACGG<br>ACCTTTGACAACGGCTCTATTCCCCATCAAATTCACTTGGGTGAGCTGCA<br>TGCTATTTTGAGAAGACAAGAAGACTTTTATCCATTTTTAAAAGACAATCGT<br>GAGAAGATTGAAAAAATCTTGACTTTTCGAATTCCTTATTATGTTGGTCCAT<br>TGGCGCGTGGCAATAGTCGTTTTGCATGGATGACTCGGAAGTCTGAAGAA<br>ACAATTACCCCATGGAATTTTGAAGAAGTTGTCGATAAAGGTGCTTCAGCT<br>CAATCATTTATTGAACGCATGACAAACTTTGATAAAAATCTTCCAAATGAAA<br>AAGTACTACCAAAACATAGTTTGCTTTATGAGTATTTTACGGTTTATAACGA<br>ATTGACAAAGGTCAAATATGTTACTGAAGGAATGCGAAAACCAGCATTTCT<br>TTCAGGTGAACAGAAGAAAGCCATTGTTGATTTACTCTTCAAAACAAATCG<br>AAAAGTAACCGTTAAGCAATTAAAAGAAGATTATTTCAAAAAAATAGAATGT<br>TTTGATAGTGTTGAAATTTCAGGAGTTGAAGATAGATTTAATGCTTCATTA<br>GGTACCTACCATGATTTGCTAAAAATTATTAAAGATAAAGATTTTTTGGATA<br>ATGAAGAAAATGAAGATATCTTAGAGGATATTGTTTTAACATTGACCTTATT<br>TGAAGATAGGGAGATGATTGAGGAAAGACTTAAAACATATGCTCACCTCT<br>TTGATGATAAGGTGATGAAACAGCTTAAACGTCGCCGTTATACTGGTTGG<br>GGACGTTTGTCTCGAAAATTGATTAATGGTATTAGGGATAAGCAATCTGG<br>CAAAACAATATTAGATTTTTTGAAATCAGATGGTTTTGCCAATCGCAATTTT<br>ATGCAGCTGATCCATGATGATAGTTTGACATTTAAAGAAGACATTCAAAAA<br>GCACAAGTGTCTGGACAAGGCGATAGTTTACATGAACATATTGCAAATTTA<br>GCTGGTAGCCCTGCTATTAAAAAAGGTATTTTACAGACTGTAAAAGTTGTT<br>GATGAATTGGTCAAAGTAATGGGGCGGCATAAGCCAGAAAATATCGTTAT<br>TGAAATGGCACGTGAAAATCAGACAACTCAAAAGGGCCAGAAAAATTCGC<br>GAGAGCGTATGAAACGAATCGAAGAAGGTATCAAAGAATTAGGAAGTCAG<br>ATTCTTAAAGAGCATCCTGTTGAAAATACTCAATTGCAAAATGAAAAGCTC<br>TATCTCTATTATCTCCAAAATGGAAGAGACATGTATGTGGACCAAGAATTA<br>GATATTAATCGTTTAAGTGATTATGATGTCGATGCCATTGTTCCACAAAGT<br>TTCCTTAAAGACGATTCAATAGACAATAAGGTCTTAACGCGTTCTGATAAA<br>AATCGTGGTAAATCGGATAACGTTCCAAGTGAAGAAGTAGTCAAAAAGAT<br>GAAAAACTATTGGAGACAACTTCTAAACGCCAAGTTAATCACTCAACGTAA<br>GTTTGATAATTTAACGAAAGCTGAACGTGGAGGTTTGAGTGAACTTGATAA<br>AGCTGGTTTTATCAAACGCCAATTGGTTGAAACTCGCCAAATCACTAAGCA<br>TGTGGCACAAATTTTGGATAGTCGCATGAATACTAAATACGATGAAAATGA<br>TAAACTTATTCGAGAGGTTAAAGTGATTACCTTAAAATCTAAATTAGTTTCT<br>GACTTCCGAAAAGATTTCCAATTCTATAAAGTACGTGAGATTAACAATTAC<br>CATCATGCCCATGATGCGTATCTAAATGCCGTCGTTGGAACTGCTTTGATT<br>AAGAAATATCCAAAACTTGAATCGGAGTTTGTCTATGGTGATTATAAAGTT<br>TATGATGTTCGTAAAATGATTGCTAAGTCTGAGCAAGAAATAGGCAAAGCA<br>ACCGCAAAATATTTCTTTTACTCTAATATCATGAACTTCTTCAAAACAGAAA<br>TTACACTTGCAAATGGAGAGATTCGCAAACGCCCTCTAATCGAAACTAAT<br>GGGGAAACTGGAGAAATTGTCTGGGATAAAGGGCGAGATTTTGCCACAG<br>TGCGCAAAGTATTGTCCATGCCCCAAGTCAATATTGTCAAGAAAACAGAA<br>GTACAGACAGGCGGATTCTCCAAGGAGTCAATTTTACCAAAAAGAAATTC<br>GGACAAGCTTATTGCTCGTAAAAAAGACTGGGATCCAAAAAAATATGGTG<br>GTTTTGATAGTCCAACGGTAGCTTATTCAGTCCTAGTGGTTGCTAAGGTG<br>GAAAAAGGGAAATCGAAGAAGTTAAAATCCGTTAAAGAGTTACTAGGGAT<br>CACAATTATGGAAAGAAGTTCCTTTGAAAAAAATCCGATTGACTTTTTAGA<br>AGCTAAAGGATATAAGGAAGTTAAAAAAGACTTAATCATTAAACTACCTAA<br>ATATAGTCTTTTTGAGTTAGAAAACGGTCGTAAACGGATGCTGGCTAGTG<br>CCGGAGAATTACAAAAAGGAAATGAGCTGGCTCTGCCAAGCAAATATGTG

	AATTTTTATATTTAGCTAGTCATTATGAAAAGTTGAAGGGTAGTCCAGAA<br>GATAACGAACAAAAACAATTGTTTGTGGAGCAGCATAAGCATTATTTAGAT<br>GAGATTATTGAGCAAATCAGTGAATTTTCTAAGCGTGTTATTTTAGCAGAT<br>GCCAATTTAGATAAAGTTCTTAGTGCATATAACAAACATAGAGACAAACCA<br>ATACGTGAACAAGCAGAAAATATTATTCATTTATTTACGTTGACGAATCTTG<br>GAGCTCCCGCTGCTTTTAAATATTTTGATACAACAATTGATCGTAAACGAT<br>ATACGTCTACAAAAGAAGTTTTAGATGCCACTCTTATCCATCAATCCATCA<br>CTGGTCTTTATGAAACACGCATTGATTTGAGTCAGCTAGGAGGTGACTAA<br>CTCGAGTAAGGATCTCCAGGCATCAAATAAAACGAAAGGCTCAGTCGAAA<br>GACTGGGCCTTTCGTTTTATCTGTTGTTTGTCGGTGAACGCTCTCTACTAG<br>AGTCACACTGGCTCACCTTCGGGTGGGCCTTTCTGCGTTTATACCTAGGG<br>ATATATTCCGCTTCCTCGCTCACTGACTCGCTACGCTCGGTCGTTCGACT<br>GCGGCGAGCGGAAATGGCTTACGAACGGGGCGGAGATTTCCTGGAAGAT<br>GCCAGGAAGATACTTAACAGGGAAGTGAGAGGGCCGCGGCAAAGCCGTT<br>TTTCCATAGGCTCCGCCCCCCTGACAAGCATCACGAAATCTGACGCTCAA<br>ATCAGTGGTGGCGAAACCCGACAGGACTATAAAGATACCAGGCGTTTCC<br>CCCTGGCGGCTCCCTCGTGCGCTCTCCTGTTCCTGCCTTTCGGTTTACCG<br>GTGTCATTCCGCTGTTATGGCCGCGTTTGTCTCATTCCACGCCTGACACT<br>CAGTTCCGGGTAGGCAGTTCGCTCCAAGCTGGACTGTATGCACGAACCC<br>CCCGTTCAGTCCGACCGCTGCGCCTTATCCGGTAACTATCGTCTTGAGTC<br>CAACCCGGAAAGACATGCAAAAGCACCACTGGCAGCAGCCACTGGTAAT<br>TGATTTAGAGGAGTTAGTCTTGAAGTCATGCGCCGGTTAAGGCTAAACTG<br>AAAGGACAAGTTTTGGTGACTGCGCTCCTCCAAGCCAGTTACCTCGGTTC<br>AAAGAGTTGGTAGCTCAGAGAACCTTCGAAAAACCGCCCTGCAAGGCGG<br>TTTTTTCGTTTTCAGAGCAAGAGATTACGCGCAGACCAAAACGATCTCAAG<br>AAGATCATCTTATTAATCAGATAAAATATTTCTAGATTTCAGTGCAATTTAT<br>CTCTTCAAATGTAGCACCTGAAGTCAGCCCCATACGATATAAGTTGTTACT<br>AGTGCTTGGATTCTCACCAATAAAAAACGCCCGGCGGCAACCGAGCGTT<br>CTGAACAAATCCAGATGGAGTTCTGAGGTCATTACTGGATCTATCAACAG<br>GAGTCCAAGCGAGCTCGATATCAAATTACGCCCCGCCCTGCCACTCATC<br>GCAGTACTGTTGTAATTCATTAAGCATTCTGCCGACATGGAAGCCATCAC<br>AAACGGCATGATGAACCTGAATCGCCAGCGGCATCAGCACCTTGTCGCC<br>TTGCGTATAATATTTGCCCATGGTGAAAACGGGGGCGAAGAAGTTGTCCA<br>TATTGGCCACGTTTAAATCAAAACTGGTGAAACTCACCCAGGGATTGGCT<br>GAGACGAAAAACATATTCTCAATAAACCCTTTAGGGAAATAGGCCAGGTTT<br>TCACCGTAACACGCCACATCTTGCGAATATATGTGTAGAAACTGCCGGAA<br>ATCGTCGTGGTATTCACTCCAGAGCGATGAAAACGTTTCAGTTTGCTCAT<br>GGAAAACGGTGTAACAAGGGTGAACACTATCCCATATCACCAGCTCACCG<br>TCTTTCATTGCCATACGAAATTCCGGATGAGCATTCATCAGGCGGGCAAG<br>AATGTGAATAAAGGCCGGATAAAACTTGTGCTTATTTTTCTTTACGGTCTT<br>TAAAAAGGCCGTAATATCCAGCTGAACGGTCTGGTTATAGGTACATTGAG<br>CAACTGACTGAAATGCCTCAAAATGTTCTTTACGATGCCATTGGGATATAT<br>CAACGGTGGTATATCCAGTGATTTTTTTCTCCATTTTAGCTTCCTTAGCTC<br>CTGAAAATCTCGATAACTCAAAAAATACGCCCGGTAGTGATCTTATTTCAT<br>TATGGTGAAAGTTGGAACCTCTTACGTGCCGATCAACGTCTCATTTTCGC<br>CAGATATC
Example Constitutive dCas9-SYNZIP	GACGTCTTTACGGCTAGCTCAGTCCTAGGTATTATGCTAGCGAATTCAAA<br>AGATCTAAAGAGGAGAAAGGATCTATGGATAAGAAATACTCAATAGGCTT<br>AGCTATCGGCACAAATAGCGTCGGATGGGCGGTGATCACTGATGAATATA<br>AGGTTCCGTCTAAAAAGTTCAAGGTTCTGGGAAATACAGACCGCCACAGT<br>ATCAAAAAAAATCTTATAGGGGCTCTTTTATTTGACAGTGGAGAGACAGCG

pJEC585<br>(Promoter-<br>RBS-<br>dCas9-<br>linker-<br>SYNZIP18-<br>Terminator-<br>p15A-Cam)	GAAGCGACTCGTCTCAAACGGACAGCTCGTAGAAGGTATACACGTCGGA<br>AGAATCGTATTTGTTATCTACAGGAGATTTTTTCAAATGAGATGGCGAAAG<br>TAGATGATAGTTTCTTTCATCGACTTGAAGAGTCTTTTTTGGTGGAAGAAG<br>ACAAGAAGCATGAACGTCATCCTATTTTTGGAAATATAGTAGATGAAGTTG<br>CTTATCATGAGAAATATCCAACTATCTATCATCTGCGAAAAAAATTGGTAG<br>ATTCTACTGATAAAGCGGATTTGCGCTTAATCTATTTGGCCTTAGCGCATA<br>TGATTAAGTTTCGTGGTCATTTTTTGATTGAGGGAGATTTAAATCCTGATA<br>ATAGTGATGTGGACAAACTATTTATCCAGTTGGTACAAACCTACAATCAAT<br>TATTTGAAGAAAACCCTATTAACGCAAGTGGAGTAGATGCTAAAGCGATTC<br>TTTCTGCACGATTGAGTAAATCAAGACGATTAGAAAATCTCATTGCTCAGC<br>TCCCCGGTGAGAAGAAAAATGGCTTATTTGGGAATCTCATTGCTTTGTCAT<br>TGGGTTTGACCCCTAATTTTAAATCAAATTTTGATTTGGCAGAAGATGCTA<br>AATTACAGCTTTCAAAAGATACTTACGATGATGATTTAGATAATTTATTGGC<br>GCAAATTGGAGATCAATATGCTGATTTGTTTTTGGCAGCTAAGAATTTATC<br>AGATGCTATTTTACTTTCAGATATCCTAAGAGTAAATACTGAAATAACTAAG<br>GCTCCCCTATCAGCTTCAATGATTAAACGCTACGATGAACATCATCAAGAC<br>TTGACTCTTTTAAAAGCTTTAGTTCGACAACAACTTCCAGAAAAGTATAAA<br>GAAATCTTTTTTGATCAATCAAAAAACGGATATGCAGGTTATATTGATGGG<br>GGAGCTAGCCAAGAAGAATTTTATAAATTTATCAAACCAATTTTAGAAAAA<br>ATGGATGGTACTGAGGAATTATTGGTGAAACTAAATCGTGAAGATTTGCT<br>GCGCAAGCAACGGACCTTTGACAACGGCTCTATTCCCCATCAAATTCACT<br>TGGGTGAGCTGCATGCTATTTTGAGAAGACAAGAAGACTTTTATCCATTTT<br>TAAAAGACAATCGTGAGAAGATTGAAAAAATCTTGACTTTTCGAATTCCTT<br>ATTATGTTGGTCCATTGGCGCGTGGCAATAGTCGTTTTGCATGGATGACT<br>CGGAAGTCTGAAGAAACAATTACCCCATGGAATTTTGAAGAAGTTGTCGA<br>TAAAGGTGCTTCAGCTCAATCATTTATTGAACGCATGACAAACTTTGATAA<br>AAATCTTCCAAATGAAAAAGTACTACCAAAACATAGTTTGCTTTATGAGTAT<br>TTTACGGTTTATAACGAATTGACAAAGGTCAAATATGTTACTGAAGGAATG<br>CGAAAACCAGCATTTCTTTCAGGTGAACAGAAGAAAGCCATTGTTGATTTA<br>CTCTTCAAAACAAATCGAAAAGTAACCGTTAAGCAATTAAAAGAAGATTAT<br>TTCAAAAAAATAGAATGTTTTGATAGTGTTGAAATTTCAGGAGTTGAAGAT<br>AGATTTAATGCTTCATTAGGTACCTACCATGATTTGCTAAAAATTATTAAAG<br>ATAAAGATTTTTTGGATAATGAAGAAAATGAAGATATCTTAGAGGATATTGT<br>TTTAACATTGACCTTATTTGAAGATAGGGAGATGATTGAGGAAAGACTTAA<br>AACATATGCTCACCTCTTTGATGATAAGGTGATGAAACAGCTTAAACGTCG<br>CCGTTATACTGGTTGGGGACGTTTGTCTCGAAAATTGATTAATGGTATTAG<br>GGATAAGCAATCTGGCAAAACAATATTAGATTTTTTGAAATCAGATGGTTT<br>TGCCAATCGCAATTTTATGCAGCTGATCCATGATGATAGTTTGACATTTAA<br>AGAAGACATTCAAAAAGCACAAGTGTCTGGACAAGGCGATAGTTTACATG<br>AACATATTGCAAATTTAGCTGGTAGCCCTGCTATTAAAAAAGGTATTTTAC<br>AGACTGTAAAAGTTGTTGATGAATTGGTCAAAGTAATGGGGCGGCATAAG<br>CCAGAAAATATCGTTATTGAAATGGCACGTGAAAATCAGACAACTCAAAAG<br>GGCCAGAAAAATTCGCGAGAGCGTATGAAACGAATCGAAGAAGGTATCAA<br>AGAATTAGGAAGTCAGATTCTTAAAGAGCATCCTGTTGAAAATACTCAATT<br>GCAAAATGAAAAGCTCTATCTCTATTATCTCCAAAATGGAAGAGACATGTA<br>TGTGGACCAAGAATTAGATATTAATCGTTTAAGTGATTATGATGTCGATGC<br>CATTGTTCCACAAAGTTTCCTTAAAGACGATTCAATAGACAATAAGGTCTT<br>AACGCGTTCTGATAAAAATCGTGGTAAATCGGATAACGTTCCAAGTGAAG<br>AAGTAGTCAAAAAGATGAAAAACTATTGGAGACAACTTCTAAACGCCAAGT<br>TAATCACTCAACGTAAGTTTGATAATTTAACGAAAGCTGAACGTGGAGGTT<br>TGAGTGAACTTGATAAAGCTGGTTTTATCAAACGCCAATTGGTTGAAACTC

	GCCAAATCACTAAGCATGTGGCACAAATTTTGGATAGTCGCATGAATACTA<br>AATACGATGAAAATGATAAACTTATTCGAGAGGTTAAAGTGATTACCTTAA<br>AATCTAAATTAGTTTCTGACTTCCGAAAAGATTTCCAATTCTATAAAGTACG<br>TGAGATTAACAATTACCATCATGCCCATGATGCGTATCTAAATGCCGTCGT<br>TGGAACTGCTTTGATTAAGAAATATCCAAAACTTGAATCGGAGTTTGTCTA<br>TGGTGATTATAAAGTTTATGATGTTCGTAAAATGATTGCTAAGTCTGAGCA<br>AGAAATAGGCAAAGCAACCGCAAAATATTTCTTTTACTCTAATATCATGAA<br>CTTCTTCAAAACAGAAATTACACTTGCAAATGGAGAGATTCGCAAACGCC<br>CTCTAATCGAAACTAATGGGGAAACTGGAGAAATTGTCTGGGATAAAGGG<br>CGAGATTTTGCCACAGTGCGCAAAGTATTGTCCATGCCCCAAGTCAATAT<br>TGTCAAGAAAACAGAAGTACAGACAGGCGGATTCTCCAAGGAGTCAATTT<br>TACCAAAAAGAAATTCGGACAAGCTTATTGCTCGTAAAAAAGACTGGGAT<br>CCAAAAAAATATGGTGGTTTTGATAGTCCAACGGTAGCTTATTCAGTCCTA<br>GTGGTTGCTAAGGTGGAAAAAGGGAAATCGAAGAAGTTAAAATCCGTTAA<br>AGAGTTACTAGGGATCACAATTATGGAAAGAAGTTCCTTTGAAAAAAATCC<br>GATTGACTTTTTAGAAGCTAAAGGATATAAGGAAGTTAAAAAAGACTTAAT<br>CATTAAACTACCTAAATATAGTCTTTTTGAGTTAGAAAACGGTCGTAAACG<br>GATGCTGGCTAGTGCCGGAGAATTACAAAAAGGAAATGAGCTGGCTCTG<br>CCAAGCAAATATGTGAATTTTTTATATTTAGCTAGTCATTATGAAAAGTTGA<br>AGGGTAGTCCAGAAGATAACGAACAAAAACAATTGTTTGTGGAGCAGCAT<br>AAGCATTATTTAGATGAGATTATTGAGCAAATCAGTGAATTTTCTAAGCGT<br>GTTATTTTAGCAGATGCCAATTTAGATAAAGTTCTTAGTGCATATAACAAAC<br>ATAGAGACAAACCAATACGTGAACAAGCAGAAAATATTATTCATTTATTTA<br>CGTTGACGAATCTTGGAGCTCCCGCTGCTTTTAAATATTTTGATACAACAA<br>TTGATCGTAAACGATATACGTCTACAAAAGAAGTTTTAGATGCCACTCTTA<br>TCCATCAATCCATCACTGGTCTTTATGAAACACGCATTGATTTGAGTCAGC<br>TAGGAGGTGACGGAGGTTCTGGAGGTGGTGGGTCCATGTCTATCGCGGC<br>CACCCTGGAAAACGACTTAGCACGCCTTGAGAATGAAAATGCTCGTCTGG<br>AGAAAGATATCGCGAATCTCGAGCGTGACCTGGCAAAATTAGAGCGTGA<br>GGAAGCTTATTTCTAACTCGAGTAAGGATCTCCAGGCATCAAATAAAACG<br>AAAGGCTCAGTCGAAAGACTGGGCCTTTCGTTTTATCTGTTGTTTGTCGG<br>TGAACGCTCTCTACTAGAGTCACACTGGCTCACCTTCGGGTGGGCCTTTC<br>TGCGTTTATACCTAGGGATATATTCCGCTTCCTCGCTCACTGACTCGCTAC<br>GCTCGGTCGTTCGACTGCGGCGAGCGGAAATGGCTTACGAACGGGGCG<br>GAGATTTCCTGGAAGATGCCAGGAAGATACTTAACAGGGAAGTGAGAGG<br>GCCGCGGCAAAGCCGTTTTTCCATAGGCTCCGCCCCCCTGACAAGCATC<br>ACGAAATCTGACGCTCAAATCAGTGGTGGCGAAACCCGACAGGACTATAA<br>AGATACCAGGCGTTTCCCCCTGGCGGCTCCCTCGTGCGCTCTCCTGTTC<br>CTGCCTTTCGGTTTACCGGTGTCATTCCGCTGTTATGGCCGCGTTTGTCT<br>CATTCCACGCCTGACACTCAGTTCCGGGTAGGCAGTTCGCTCCAAGCTG<br>GACTGTATGCACGAACCCCCCGTTCAGTCCGACCGCTGCGCCTTATCCG<br>GTAACTATCGTCTTGAGTCCAACCCGGAAAGACATGCAAAAGCACCACTG<br>GCAGCAGCCACTGGTAATTGATTTAGAGGAGTTAGTCTTGAAGTCATGCG<br>CCGGTTAAGGCTAAACTGAAAGGACAAGTTTTGGTGACTGCGCTCCTCCA<br>AGCCAGTTACCTCGGTTCAAAGAGTTGGTAGCTCAGAGAACCTTCGAAAA<br>ACCGCCCTGCAAGGCGGTTTTTTCGTTTTCAGAGCAAGAGATTACGCGCA<br>GACCAAAACGATCTCAAGAAGATCATCTTATTAATCAGATAAAATATTTCTA<br>GATTTCAGTGCAATTTATCTCTTCAAATGTAGCACCTGAAGTCAGCCCCAT<br>ACGATATAAGTTGTTACTAGTGCTTGGATTCTCACCAATAAAAACGCCCG<br>GCGGCAACCGAGCGTTCTGAACAAATCCAGATGGAGTTCTGAGGTCATTA<br>CTGGATCTATCAACAGGAGTCCAAGCGAGCTCGATATCAAATTACGCCCC

	GCCCTGCCACTCATCGCAGTACTGTTGTAATTCATTAAGCATTCTGCCGA<br>CATGGAAGCCATCACAAACGGCATGATGAACCTGAATCGCCAGCGGCAT<br>CAGCACCTTGTCGCCTTGCGTATAATATTTGCCCATGGTGAAAACGGGGG<br>CGAAGAAGTTGTCCATATTGGCCACGTTTAAATCAAAACTGGTGAAACTCA<br>CCCAGGGATTGGCTGAGACGAAAAACATATTCTCAATAAACCCTTTAGGG<br>AAATAGGCCAGGTTTTCACCGTAACACGCCACATCTTGCGAATATATGTG<br>TAGAAACTGCCGGAAATCGTCGTGGTATTCACTCCAGAGCGATGAAAACG<br>TTTCAGTTTGCTCATGGAAAACGGTGTAACAAGGGTGAACACTATCCCAT<br>ATCACCAGCTCACCGTCTTTCATTGCCATACGAAATTCCGGATGAGCATT<br>CATCAGGCGGGCAAGAATGTGAATAAAGGCCGGATAAAACTTGTGCTTAT<br>TTTTCTTTACGGTCTTTAAAAAGGCCGTAATATCCAGCTGAACGGTCTGGT<br>TATAGGTACATTGAGCAACTGACTGAAATGCCTCAAAATGTTCTTTACGAT<br>GCCATTGGGATATATCAACGGTGGTATATCCAGTGATTTTTTTCTCCATTT<br>TAGCTTCCTTAGCTCCTGAAAATCTCGATAACTCAAAAAATACGCCCGGTA<br>GTGATCTTATTTCATTATGGTGAAAGTTGGAACCTCTTACGTGCCGATCAA<br>CGTCTCATTTTCGCCAGATATC
Example RFP reporter plasmid pJEC581 (KanR- PAM rich sequence – promoter – RBS- RFP- Terminator – Rep101	GTCTGACCACTTCGGATTATCCCGTGACAGGTCATTCAGACTGGCTAATG<br>CACCCAGTAAGGCAGCGGTATCATCAACAGGCTTACCCGTCTTACTGTTG<br>TTACAACCAATTAACCAATTCTGATTAGAAAAACTCATCGAGCATCAAATG<br>AAACTGCAATTTATTCATATCAGGATTATCAATACCATATTTTTGAAAAAGC<br>CGTTTCTGTAATGAAGGAGAAAACTCACCGAGGCAGTTCCATAGGATGGC<br>AAGATCCTGGTATCGGTCTGCGATTCCGACTCGTCCAACATCAATACAAC<br>CTATTAATTTCCCCTCGTCAAAAATAAGGTTATCAAGTGAGAAATCACCAT<br>GAGTGACGACTGAATCCGGTGAGAATGGCAAAAGCTTATGCATTTCTTTC<br>CAGACTTGTTCAACAGGCCAGCCATTACGCTCGTCATCAAAATCACTCGC<br>ATCAACCAAACCGTTATTCATTCGTGATTGCGCCTGAGCGAGGCGAAATA<br>CGCGATCGCTGTTAAAAGGACAATTACAAACAGGAATCGAATGCAACCGG<br>CGCAGGAACACTGCCAGCGCATCAACAATATTTTCACCTGAATCAGGATA<br>TTCTTCTAATACCTGGAATGCTGTTTTCCCGGGGATCGCAGTGGTGAGTA<br>ACCATGCATCATCAGGAGTACGGATAAAATGCTTGATGGTCGGAAGAGGC<br>ATAAATTCCGTCAGCCAGTTTAGTCTGACCATCTCATCTGTAACATCATTG<br>GCAACGCTACCTTTGCCATGTTTCAGAAACAACTCTGGCGCATCGGGCTT<br>CCCATACAATCGATAGATTGTCGCACCTGATTGCCCGACATTATCGCGAG<br>CCCATTTATACCCATATAAATCAGCATCCATGTTGGAATTTAATCGCGGCC<br>TGGAGCAAGACGTTTCCCGTTGAATATGGCTCATAACACCCCTTGTATTA<br>CTGTTTATGTAAGCAGACAGTTTTATTGTTCATGATGATATATTTTTATCTT<br>GTGCAATGTAACATCAGAGATTTTGAGACACAACGTGGCTTTGTTGAATAA<br>ATCGAACTTTTGCTGAGTTGAAGGATCAGCACATTTCCCCGAAAAGTGCC<br>ACCTGTGGCAATTCCGACGTCGCCTACGGTATCCACCGGAGACCTATGG<br>CAGCCTCCGGCCGCCATAGGACACCTTTGGTTGCCAAGGGTGACCTATG<br>GTGACCATGGGCCACCACGGGCGACCTCAGGTATCCTGCGGTGTCCTGC<br>GGTTACCAAAGGCGTCCTTTGGGTTCCACCGGATACCTCCGGACTTGACA<br>GCTAGCTCAGTCCTAGGGATTGTGCTAGCGAATTCATTAAGAGGAGAAAA<br>AGAGGAGAAAGGTACATGGCGAGTAGCGAAGACGTTATCAAAGAGTTCAT<br>GCGTTTCAAAGTTCGTATGGAAGGTTCCGTTAACGGTCACGAGTTCGAAA<br>TCGAAGGTGAAGGTGAAGGTCGTCCGTACGAAGGTACCCAGACCGCTAA<br>ACTGAAAGTTACCAAAGGTGGTCCGCTGCCGTTCGCTTGGGACATCCTGT<br>CCCCGCAGTTCCAGTACGGTTCCAAAGCTTACGTTAAACACCCGGCTGAC<br>ATCCCGGACTACCTGAAACTGTCCTTCCCGGAAGGTTTCAAATGGGAACG<br>TGTTATGAACTTCGAAGACGGTGGTGTTGTTACCGTTACCCAGGACTCCT<br>CCCTGCAAGACGGTGAGTTCATCTACAAAGTTAAACTGCGTGGTACCAAC

	TTCCCGTCCGACGGTCCGGTTATGCAGAAAAAAACCATGGGTTGGGAAG<br>CTTCCACCGAACGTATGTACCCGGAAGACGGTGCTCTGAAAGGTGAAATC<br>AAAATGCGTCTGAAACTGAAAGACGGTGGTCACTACGACGCTGAAGTTAA<br>AACCACCTACATGGCTAAAAAACCGGTTCAGCTGCCGGGTGCTTACAAAA<br>CCGACATCAAACTGGACATCACCTCCCACAACGAAGACTACACCATCGTT<br>GAACAGTACGAACGTGCTGAAGGTCGTCACTCCACCGGTGCTTAAGGAT<br>CCAAACTCGAGTAAGGATCTCCAGGCATCAAATAAAACGAAAGGCTCAGT<br>CGAAAGACTGGGCCTTTCGTTTTATCTGTTGTTTGTCGGTGAACGCTCTCT<br>ACTAGAGTCACACTGGCTCACCTTCGGGTGGGCCTTTCTGCGTTTATACC<br>TAGGGTACGGGTTTTGCTGCCCGCAAACGGGCTGTTCTGGTGTTGCTAG<br>TTTGTTATCAGAATCGCAGATCCGGCTTCAGCCGGTTTGCCGGCTGAAAG<br>CGCTATTTCTTCCAGAATTGCCATGATTTTTTCCCCACGGGAGGCGTCAC<br>TGGCTCCCGTGTTGTCGGCAGCTTTGATTCGATAAGCAGCATCGCCTGTT<br>TCAGGCTGTCTATGTGTGACTGTTGAGCTGTAACAAGTTGTCTCAGGTGT<br>TCAATTTCATGTTCTAGTTGCTTTGTTTTACTGGTTTCACCTGTTCTATTAG<br>GTGTTACATGCTGTTCATCTGTTACATTGTCGATCTGTTCATGGTGAACAG<br>CTTTGAATGCACCAAAAACTCGTAAAAGCTCTGATGTATCTATCTTTTTTAC<br>ACCGTTTTCATCTGTGCATATGGACAGTTTTCCCTTTGATATGTAACGGTG<br>AACAGTTGTTCTACTTTTGTTTGTTAGTCTTGATGCTTCACTGATAGATACA<br>AGAGCCATAAGAACCTCAGATCCTTCCGTATTTAGCCAGTATGTTCTCTAG<br>TGTGGTTCGTTGTTTTTGCGTGAGCCATGAGAACGAACCATTGAGATCAT<br>ACTTACTTTGCATGTCACTCAAAAATTTTGCCTCAAAACTGGTGAGCTGAA<br>TTTTTGCAGTTAAAGCATCGTGTAGTGTTTTTCTTAGTCCGTTATGTAGGT<br>AGGAATCTGATGTAATGGTTGTTGGTATTTTGTCACCATTCATTTTTATCTG<br>GTTGTTCTCAAGTTCGGTTACGAGATCCATTTGTCTATCTAGTTCAACTTG<br>GAAAATCAACGTATCAGTCGGGCGGCCTCGCTTATCAACCACCAATTTCA<br>TATTGCTGTAAGTGTTTAAATCTTTACTTATTGGTTTCAAAACCCATTGGTT<br>AAGCCTTTTAAACTCATGGTAGTTATTTTCAAGCATTAACATGAACTTAAAT<br>TCATCAAGGCTAATCTCTATATTTGCCTTGTGAGTTTTCTTTTGTGTTAGTT<br>CTTTTAATAACCACTCATAAATCCTCATAGAGTATTTGTTTTCAAAAGACTT<br>AACATGTTCCAGATTATATTTTATGAATTTTTTTAACTGGAAAAGATAAGGC<br>AATATCTCTTCACTAAAAACTAATTCTAATTTTTCGCTTGAGAACTTGGCAT<br>AGTTTGTCCACTGGAAAATCTCAAAGCCTTTAACCAAAGGATTCCTGATTT<br>CCACAGTTCTCGTCATCAGCTCTCTGGTTGCTTTAGCTAATACACCATAAG<br>CATTTTCCCTACTGATGTTCATCATCTGAGCGTATTGGTTATAAGTGAACG<br>ATACCGTCCGTTCTTTCCTTGTAGGGTTTTCAATCGTGGGGTTGAGTAGT<br>GCCACACAGCATAAAATTAGCTTGGTTTCATGCTCCGTTAAGTCATAGCG<br>ACTAATCGCTAGTTCATTTGCTTTGAAAACAACTAATTCAGACATACATCTC<br>AATTGGTCTAGGTGATTTTAATCACTATACCAATTGAGATGGGCTAGTCAA<br>TGATAATTACTAGTCCTTTTCCCGGGTGATCTGGGTATCTGTAAATTCTGC<br>TAGACCTTTGCTGGAAAACTTGTAAATTCTGCTAGACCCTCTGTAAATTCC<br>GCTAGACCTTTGTGTGTTTTTTTTGTTTATATTCAAGTGGTTATAATTTATA<br>GAATAAAGAAAGAATAAAAAAAGATAAAAAGAATAGATCCCAGCCCTGTGT<br>ATAACTCACTACTTTAGTCAGTTCCGCAGTATTACAAAAGGATGTCGCAAA<br>CGCTGTTTGCTCCTCTACAAAACAGACCTTAAAACCCTAAAGGCTTAAGTA<br>GCACCCTCGCAAGCTCGGGCAAATCGCTGAATATTCCTTTTGTCTCCGAC<br>CATCAGGCACCTGAGTCGCTGTCTTTTTCGTGACATTCAGTTCGCTGCGC<br>TCACGGCTCTGGCAGTGAATGGGGGTAAATGGCACTACAGGCGCCTTTT<br>ATGGATTCATGCAAGGAAACTACCCATAATACAAGAAAAGCCCGTCACGG<br>GCTTCTCAGGGCGTTTTATGGCGGGTCTGCTATGTGGTGCTATCTGACTT<br>TTTGCTGTTCAGCAGTTCCTGCCCTCTGATTTTCCA

Example sgRNA plasmid pJEC566 (Promoter – sgRNA spacer – sgRNA hairpins– Terminator – ColE1 - AmpR	GAATTCTAAAGATCTTTGACAGCTAGCTCAGTCCTAGGTATAATACTAGTA<br>GGACGCCTTTGGTAACCGCGTTTTAGAGCTAGAAATAGCAAGTTAAAATA<br>AGGCTAGTCCGTTATCAACTTGAAAAAGTGGCACCGAGTCGGTGCTTTTT<br>TTGAAGCTTGGGCCCGAACAAAAACTCATCTCAGAAGAGGATCTGAATAG<br>CGCCGTCGACCATCATCATCATCATCATTGAGTTTAAACGGTCTCCAGCTT<br>GGCTGTTTTGGCGGATGAGAGAAGATTTTCAGCCTGATACAGATTAAATC<br>AGAACGCAGAAGCGGTCTGATAAAACAGAATTTGCCTGGCGGCAGTAGC<br>GCGGTGGTCCCACCTGACCCCATGCCGAACTCAGAAGTGAAACGCCGTA<br>GCGCCGATGGTAGTGTGGGGTCTCCCCATGCGAGAGTAGGGAACTGCCA<br>GGCATCAAATAAAACGAAAGGCTCAGTCGAAAGACTGGGCCTTTCGTTTT<br>ATCTGTTGTTTGTCGGTGAACTGGATCCTTACTCGAGTCTAGACTGCAGG<br>CTTCCTCGCTCACTGACTCGCTGCGCTCGGTCGTTCGGCTGCGGCGAGC<br>GGTATCAGCTCACTCAAAGGCGGTAATACGGTTATCCACAGAATCAGGGG<br>ATAACGCAGGAAAGAACATGTGAGCAAAAGGCCAGCAAAAGGCCAGGAA<br>CCGTAAAAAGGCCGCGTTGCTGGCGTTTTTCCATAGGCTCCGCCCCCCT<br>GACGAGCATCACAAAAATCGACGCTCAAGTCAGAGGTGGCGAAACCCGA<br>CAGGACTATAAAGATACCAGGCGTTTCCCCCTGGAAGCTCCCTCGTGCG<br>CTCTCCTGTTCCGACCCTGCCGCTTACCGGATACCTGTCCGCCTTTCTCC<br>CTTCGGGAAGCGTGGCGCTTTCTCATAGCTCACGCTGTAGGTATCTCAGT<br>TCGGTGTAGGTCGTTCGCTCCAAGCTGGGCTGTGTGCACGAACCCCCCG<br>TTCAGCCCGACCGCTGCGCCTTATCCGGTAACTATCGTCTTGAGTCCAAC<br>CCGGTAAGACACGACTTATCGCCACTGGCAGCAGCCACTGGTAACAGGA<br>TTAGCAGAGCGAGGTATGTAGGCGGTGCTACAGAGTTCTTGAAGTGGTG<br>GCCTAACTACGGCTACACTAGAAGGACAGTATTTGGTATCTGCGCTCTGC<br>TGAAGCCAGTTACCTTCGGAAAAAGAGTTGGTAGCTCTTGATCCGGCAAA<br>CAAACCACCGCTGGTAGCGGTGGTTTTTTTGTTTGCAAGCAGCAGATTAC<br>GCGCAGAAAAAAAGGATCTCAAGAAGATCCTTTGATCTTTTCTACGGGGT<br>CTGACGCTCAGTGGAACGAAAACTCACGTTAAGGGATTTTGGTCATGAGA<br>TTATCAAAAAGGATCTTCACCTAGATCCTTTTAAATTAAAAATGAAGTTTTA<br>AATCAATCTAAAGTATATATGAGTAAACTTGGTCTGACAGTTACCAATGCT<br>TAATCAGTGAGGCACCTATCTCAGCGATCTGTCTATTTCGTTCATCCATAG<br>TTGCCTGACTCCCCGTCGTGTAGATAACTACGATACGGGAGGGCTTACCA<br>TCTGGCCCCAGTGCTGCAATGATACCGCGAGACCCACGCTCACCGGCTC<br>CAGATTTATCAGCAATAAACCAGCCAGCCGGAAGGGCCGAGCGCAGAAG<br>TGGTCCTGCAACTTTATCCGCCTCCATCCAGTCTATTAATTGTTGCCGGG<br>AAGCTAGAGTAAGTAGTTCGCCAGTTAATAGTTTGCGCAACGTTGTTGCC<br>ATTGCTACAGGCATCGTGGTGTCACGCTCGTCGTTTGGTATGGCTTCATT<br>CAGCTCCGGTTCCCAACGATCAAGGCGAGTTACATGATCCCCCATGTTGT<br>GCAAAAAAGCGGTTAGCTCCTTCGGTCCTCCGATCGTTGTCAGAAGTAAG<br>TTGGCCGCAGTGTTATCACTCATGGTTATGGCAGCACTGCATAATTCTCTT<br>ACTGTCATGCCATCCGTAAGATGCTTTTCTGTGACTGGTGAGTACTCAAC<br>CAAGTCATTCTGAGAATAGTGTATGCGGCGACCGAGTTGCTCTTGCCCGG<br>CGTCAATACGGGATAATACCGCGCCACATAGCAGAACTTTAAAAGTGCTC<br>ATCATTGGAAAACGTTCTTCGGGGCGAAAACTCTCAAGGATCTTACCGCT<br>GTTGAGATCCAGTTCGATGTAACCCACTCGTGCACCCAACTGATCTTCAG<br>CATCTTTTACTTTCACCAGCGTTTCTGGGTGAGCAAAAACAGGAAGGCAA<br>AATGCCGCAAAAAAGGGAATAAGGGCGACACGGAAATGTTGAATACTCAT<br>ACTCTTCCTTTTTCAATATTATTGAAGCATTTATCAGGGTTATTGTCTCATG<br>AGCGGATACATATTTGAA
Example Activator-	TTTACGGCTAGCTCAGTCCTAGGTATAGTGCTAGCCGCTTCTAGAGTCAC<br>ACAGGAAAGTACTAGATGCAGGGTTCTGTGACAGAGTTTCTAAAACCGCG

SYNZIP plasmid pJEC626 (Promoter – RBS – Activator – Linker – SYNZIP17 – Terminator – SpecR – CDF13	CCTGGTTGATATCGAGCAAGTGAGTTCGACGCACGCCAAGGTGACCCTT<br>GAGCCTTTAGAGCGTGGCTTTGGCCATACTCTGGGTAACGCACTGCGCC<br>GTATTCTGCTCTCATCGATGCCGGGTTGCGCGGTGACCGAGGTTGAGAT<br>TGATGGTGTACTACATGAGTACAGCACCAAAGAAGGCGTTCAGGAAGATA<br>TCCTGGAAATCCTGCTCAACCTGAAAGGGCTGGCGGTGAGAGTTCAGGG<br>CAAAGATGAAGTTATTCTTACCTTGAATAAATCTGGCATTGGCCCTGTGAC<br>TGCAGCCGATATCACCCACGACGGTGATGTCGAAATCGTCAAGCCGCAG<br>CACGTGATCTGCCACCTGACCGATGAGAACGCGTCTATTAGCATGCGTAT<br>CAAAGTTCAGCGCGGTCGTGGTTATGTGCCGGCTTCTACCCGAATTCATT<br>CGGAAGAAGATGAGCGCCCAATCGGCCGTCTGCTGGTCGACGCATGCTA<br>CAGCCCTGTGGAGCGTATTGCCTACAATGTTGAAGCAGCGCGTGTAGAA<br>CAGCGTACCGACCTGGACAAGCTGGTCATCGAAATGGAAACCAACGGCA<br>CAATCGATCCTGAAGAGGCGATTCGTCGTGCGGCAACCATTCTGGCTGA<br>ACAACTGGAAGCTTTCGTTGACTTACGTGATGTACGTCAGCCTGAAGTGA<br>AAGAAGAGAAACCAGAGGGAGGTTCTGGAGGTGGTGGGTCCATGAACGA<br>AAAAGAAGAACTGAAATCTAAAAAAGCGGAACTGCGCAACCGCATCGAAC<br>AGCTGAAACAGAAACGCGAACAGCTGAAACAGAAAATCGCGAACCTGCG<br>CAAAGAAATCGAAGCGTATAAATAATGACCGAACAAAAACTCATCTCAGAA<br>GAGGATCTGAATAGCGCCGTCGACCATCATCATCATCATCATTGAGTTTA<br>AACGGTCTCCAGCTTGGCTGTTTTGGCGGATGAGAGAAGATTTTCAGCCT<br>GATACAGATTAAATCAGAACGCAGAAGCGGTCTGATAAAACAGAATTTGC<br>CTGGCGGCAGTAGCGCGGTGGTCCCACCTGACCCCATGCCGAACTCAGA<br>AGTGAAACGCCGTAGCGCCGATGGTAGTGTGGGGTCTCCCCATGCGAGA<br>GTAGGGAACTGCCAGGCATCAAATAAAACGAAAGGCTCAGTCGAAAGACT<br>GGGCCTTTCGTTTTATCTGTTGTTTGTCGGTGAACTGGATCCTTACTCGAG<br>TCTAGACTGCAGCTGAAACCTCAGGCATTTGAGAAGCACACGGTCACACT<br>GCTTCCGGTAGTCAATAAACCGGTAAACCAGCAATAGACATAAGCGGCTA<br>TTTAACGACCCTGCCCTGAACCGACGACCGGGTCATCGTGGCCGGATCT<br>TGCGGCCCCTCGGCTTGAACGAATTGTTAGACATTATTTGCCGACTACCT<br>TGGTGATCTCGCCTTTCACGTAGTGGACAAATTCTTCCAACTGATCTGCG<br>CGCGAGGCCAAGCGATCTTCTTCTTGTCCAAGATAAGCCTGTCTAGCTTC<br>AAGTATGACGGGCTGATACTGGGCCGGCAGGCGCTCCATTGCCCAGTCG<br>GCAGCGACATCCTTCGGCGCGATTTTGCCGGTTACTGCGCTGTACCAAAT<br>GCGGGACAACGTAAGCACTACATTTCGCTCATCGCCAGCCCAGTCGGGC<br>GGCGAGTTCCATAGCGTTAAGGTTTCATTTAGCGCCTCAAATAGATCCTG<br>TTCAGGAACCGGATCAAAGAGTTCCTCCGCCGCTGGACCTACCAAGGCA<br>ACGCTATGTTCTCTTGCTTTTGTCAGCAAGATAGCCAGATCAATGTCGATC<br>GTGGCTGGCTCGAAGATACCTGCAAGAATGTCATTGCGCTGCCATTCTCC<br>AAATTGCAGTTCGCGCTTAGCTGGATAACGCCACGGAATGATGTCGTCGT<br>GCACAACAATGGTGACTTCTACAGCGCGGAGAATCTCGCTCTCTCCAGG<br>GGAAGCCGAAGTTTCCAAAAGGTCGTTGATCAAAGCTCGCCGCGTTGTTT<br>CATCAAGCCTTACGGTCACCGTAACCAGCAAATCAATATCACTGTGTGGC<br>TTCAGGCCGCCATCCACTGCGGAGCCGTACAAATGTACGGCCAGCAACG<br>TCGGTTCGAGATGGCGCTCGATGACGCCAACTACCTCTGATAGTTGAGTC<br>GATACTTCGGCGATCACCGCTTCCCTCATACTCTTCCTTTTTCAATATTATT<br>GAAGCATTTATCAGGGTTATTGTCTCATGAGCGGATACATATTTGAATGTA<br>TTTAGAAAAATAAACAAATAGCTAGCTCACTCGGTCGCTACGCTCCGGGC<br>GTGAGACTGCGGCGGGCGCTGCGGACACATACAAAGTTACCCACAGATT<br>CCGTGGATAAGCAGGGGACTAACATGTGAGGCAAAACAGCAGGGCCGC<br>GCCGGTGGCGTTTTTCCATAGGCTCCGCCCTCCTGCCAGAGTTCACATAA<br>ACAGACGCTTTTCCGGTGCATCTGTGGGAGCCGTGAGGCTCAACCATGA

	ATCTGACAGTACGGGCGAAACCCGACAGGACTTAAAGATCCCCACCGTTT<br>CCGGCGGGTCGCTCCCTCTTGCGCTCTCCTGTTCCGACCCTGCCGTTTA<br>CCGGATACCTGTTCCGCCTTTCTCCCTTACGGGAAGTGTGGCGCTTTCTC<br>ATAGCTCACACACTGGTATCTCGGCTCGGTGTAGGTCGTTCGCTCCAAGC<br>TGGGCTGTAAGCAAGAACTCCCCGTTCAGCCCGACTGCTGCGCCTTATC<br>CGGTAACTGTTCACTTGAGTCCAACCCGGAAAAGCACGGTAAAACGCCAC<br>TGGCAGCAGCCATTGGTAACTGGGAGTTCGCAGAGGATTTGTTTAGCTAA<br>ACACGCGGTTGCTCTTGAAGTGTGCGCCAAAGTCCGGCTACACTGGAAG<br>GACAGATTTGGTTGCTGTGCTCTGCGAAAGCCAGTTACCACGGTTAAGCA<br>GTTCCCCAACTGACTTAACCTTCGATCAAACCACCTCCCCAGGTGGTTTTT<br>TCGTTTACAGGGCAAAAGATTACGCGCAGAAAAAAAGGATCTCAAGAAGA<br>TCCTTTGATCTTTTCTACTGAACCGCTCTAGATTTCAGTGCAATTTATCTCT<br>TCAAATGTAGCACCTG

Supplementary Table 3. Activator domain (AD) sequences used in this study. Plasmid sequences can be constructed by replacing the light green region in the example dCas9 fusion or Activator-SYNZIP plasmids shown in **Supplementary Table 2**.

Name	DNA sequence	Protein sequence	Plasmids
ω	ATGGCACGCGTAACTGTTCAGGACGCTGTAGA<br>GAAAATTGGTAACCGTTTTGACCTGGTACTGGT<br>CGCCGCGCGTCGCGCTCGTCAGATGCAGGTAG<br>GCGGAAAGGATCCGCTGGTACCGGAAGAAAAC<br>GATAAAACCACTGTAATCGCGCTGCGCGAAATC<br>GAAGAAGGTCTGATCAACAACCAGATCCTCGAC<br>GTTCGCGAACGCCAGGAACAGCAAGAGCAGGA<br>AGCCGCTGAATTACAAGCCGTTACCGCTATTGC<br>TGAAGGTCGTCGT	MARVTVQDAV<br>EKIGNRFDLVL<br>VAARRARQM<br>QVGGKDPLVP<br>EENDKTTVIAL<br>REIEEGLINNQI<br>LDVRERQEQQ<br>EQEAAELQAV<br>TAIAEGRR	pJEC547,<br>pJEC548
α	ATGCAGGGTTCTGTGACAGAGTTTCTAAAACCG<br>CGCCTGGTTGATATCGAGCAAGTGAGTTCGAC<br>GCACGCCAAGGTGACCCTTGAGCCTTTAGAGC<br>GTGGCTTTGGCCATACTCTGGGTAACGCACTGC<br>GCCGTATTCTGCTCTCATCGATGCCGGGTTGCG<br>CGGTGACCGAGGTTGAGATTGATGGTGTACTAC<br>ATGAGTACAGCACCAAAGAAGGCGTTCAGGAA<br>GATATCCTGGAAATCCTGCTCAACCTGAAAGGG<br>CTGGCGGTGAGAGTTCAGGGCAAAGATGAAGT<br>TATTCTTACCTTGAATAAATCTGGCATTGGCCCT<br>GTGACTGCAGCCGATATCACCCACGACGGTGA<br>TGTCGAAATCGTCAAGCCGCAGCACGTGATCTG<br>CCACCTGACCGATGAGAACGCGTCTATTAGCAT<br>GCGTATCAAAGTTCAGCGCGGTCGTGGTTATGT<br>GCCGGCTTCTACCCGAATTCATTCGGAAGAAGA<br>TGAGCGCCCAATCGGCCGTCTGCTGGTCGACG<br>CATGCTACAGCCCTGTGGAGCGTATTGCCTACA<br>ATGTTGAAGCAGCGCGTGTAGAACAGCGTACC<br>GACCTGGACAAGCTGGTCATCGAAATGGAAAC	MQGSVTEFLK<br>PRLVDIEQVSS<br>THAKVTLEPLE<br>RGFGHTLGNA<br>LRRILLSSMPG<br>CAVTEVEIDGV<br>LHEYSTKEGV<br>QEDILEILLNLK<br>GLAVRVQGKD<br>EVILTLNKSGI<br>GPVTAADITHD<br>GDVEIVKPQH<br>VICHLTDENAS<br>ISMRIKVQRGR<br>GYVPASTRIHS<br>EEDERPIGRLL<br>VDACYSPVERI<br>AYNVEAARVE<br>QRTDLDKLVIE	pJEC549,<br>pJEC550

	CAACGGCACAATCGATCCTGAAGAGGCGATTC<br>GTCGTGCGGCAACCATTCTGGCTGAACAACTG<br>GAAGCTTTCGTTGACTTACGTGATGTACGTCAG<br>CCTGAAGTGAAAGAAGAGAAACCAGAGTTCGAT<br>CCGATCCTGCTGCGCCCTGTTGACGATCTGGA<br>ATTGACTGTCCGCTCTGCTAACTGCCTTAAAGC<br>AGAAGCTATCCACTATATCGGTGATCTGGTACA<br>GCGTACCGAGGTTGAGCTCCTTAAAACGCCTAA<br>CCTTGGTAAAAAATCTCTTACTGAGATTAAAGAC<br>GTGCTGGCTTCCCGTGGACTGTCTCTGGGCAT<br>GCGCCTGGAAAACTGGCCACCGGCAAGCATCG<br>CTGACGAG	METNGTIDPE<br>EAIRRAATILAE<br>QLEAFVDLRD<br>VRQPEVKEEK<br>PEFDPILLRPV<br>DDLELTVRSA<br>NCLKAEAIHYI<br>GDLVQRTEVE<br>LLKTPNLGKKS<br>LTEIKDVLASR<br>GLSLGMRLEN<br>WPPASIADE	
αNTD	ATGCAGGGTTCTGTGACAGAGTTTCTAAAACCG<br>CGCCTGGTTGATATCGAGCAAGTGAGTTCGAC<br>GCACGCCAAGGTGACCCTTGAGCCTTTAGAGC<br>GTGGCTTTGGCCATACTCTGGGTAACGCACTGC<br>GCCGTATTCTGCTCTCATCGATGCCGGGTTGCG<br>CGGTGACCGAGGTTGAGATTGATGGTGTACTAC<br>ATGAGTACAGCACCAAAGAAGGCGTTCAGGAA<br>GATATCCTGGAAATCCTGCTCAACCTGAAAGGG<br>CTGGCGGTGAGAGTTCAGGGCAAAGATGAAGT<br>TATTCTTACCTTGAATAAATCTGGCATTGGCCCT<br>GTGACTGCAGCCGATATCACCCACGACGGTGA<br>TGTCGAAATCGTCAAGCCGCAGCACGTGATCTG<br>CCACCTGACCGATGAGAACGCGTCTATTAGCAT<br>GCGTATCAAAGTTCAGCGCGGTCGTGGTTATGT<br>GCCGGCTTCTACCCGAATTCATTCGGAAGAAGA<br>TGAGCGCCCAATCGGCCGTCTGCTGGTCGACG<br>CATGCTACAGCCCTGTGGAGCGTATTGCCTACA<br>ATGTTGAAGCAGCGCGTGTAGAACAGCGTACC<br>GACCTGGACAAGCTGGTCATCGAAATGGAAAC<br>CAACGGCACAATCGATCCTGAAGAGGCGATTC<br>GTCGTGCGGCAACCATTCTGGCTGAACAACTG<br>GAAGCTTTCGTTGACTTACGTGATGTACGTCAG<br>CCTGAAGTGAAAGAAGAGAAACCAGAG	MQGSVTEFLK<br>PRLVDIEQVSS<br>THAKVTLEPLE<br>RGFGHTLGNA<br>LRRILLSSMPG<br>CAVTEVEIDGV<br>LHEYSTKEGV<br>QEDILEILLNLK<br>GLAVRVQGKD<br>EVILTLNKSGI<br>GPVTAADITHD<br>GDVEIVKPQH<br>VICHLTDENAS<br>ISMRIKVQRGR<br>GYVPASTRIHS<br>EEDERPIGRLL<br>VDACYSPVERI<br>AYNVEAARVE<br>QRTDLDKLVIE<br>METNGTIDPE<br>EAIRRAATILAE<br>QLEAFVDLRD<br>VRQPEVKEEK<br>PE	pJEC551,<br>pJEC552,<br>pJEC626
LuxR	ATGAAAAACATAAATGCCGACGACACATACAGA<br>ATAATTAATAAAATTAAAGCTTGTAGAAGCAATA<br>ATGATATTAATCAATGCTTATCTGATATGACTAA<br>AATGGTACATTGTGAATATTATTTACTCGCGATC<br>ATTTATCCTCATTCTATGGTTAAATCTGATATTTC<br>AATCCTAGATAATTACCCTAAAAAATGGAGGCA<br>ATATTATGATGACGCTAATTTAATAAAATATGAT<br>CCTATAGTAGATTATTCTAACTCCAATCATTCAC<br>CAATTAATTGGAATATATTTGAAAACAATGCTGT<br>AAATAAAAAATCTCCAAATGTAATTAAAGAAGCG<br>AAAACATCAGGTCTTATCACTGGGTTTAGTTTCC<br>CTATTCATACGGCTAACAATGGCTTCGGAATGC<br>TTAGTTTTGCACATTCAGAAAAAGACAACTATAT<br>AGATAGTTTATTTTTACATGCGTGTATGAACATA<br>CCATTAATTGTTCCTTCTCTAGTTGATAATTATC	MKNINADDTY<br>RIINKIKACRSN<br>NDINQCLSDM<br>TKMVHCEYYL<br>LAIIYPHSMVK<br>SDISILDNYPK<br>KWRQYYDDA<br>NLIKYDPIVDY<br>SNSNHSPINW<br>NIFENNAVNKK<br>SPNVIKEAKTS<br>GLITGFSFPIHT<br>ANNGFGMLSF<br>AHSEKDNYIDS<br>LFLHACMNIPLI	pJEC645,<br>pJEC646

	GAAAAATAAATATAGCAAATAATAAATCAAACAA CGATTTAACCAAAAGAGAAAAAGAATGTTTAGC GTGGGCATGCGAAGGAAAAAGCTCTTGGGATA TTTCAAAAATATTAGGTTGCAGTGAGCGTACTGT CACTTTCCATTTAACCAATGCGCAAATGAAACTC AATACAACAAACCGCTGCCAAAGTATTTCTAAA GCAATTTTAACAGGAGCAATTGATTGCCCATAC TTTAAAAAT	VPSLVDNYRKI NIANNKSNNDL TKREKECLAW ACEGKSSWDI SKILGCSERTV TFHLTNAQMK LNTTNRCQSIS KAILTGAIDCP YFKN	
SoxS	ATGTCCCATCAGAAAATTATTCAGGATCTTATCG CATGGATTGACGAGCATATTGACCAGCCGCTTA ACATTGATGTAGTCGCAAAAAAATCAGGCTATT CAAAGTGGTACTTGCAACGAATGTTCCGCACGG TGACGCATCAGACGCTTGGCGATTACATTCGCC AACGCCGCCTGTTACTGGCCGCCGTTGAGTTG CGCACCACCGAGCGTCCGATTTTTGATATCGCA ATGGACCTGGGTTATGTCTCGCAGCAGACCTTC TCCCGCGTTTTCCGTCGGCAGTTTGATCGCACT CCCAGCGATTATCGCCACCGCCTG	MSHQKIIQDLI AWIDEHIDQPL NIDVVAKKSGY SKWYLQRMFR TVTHQTLGDYI RQRRLLLAAV ELRTTERPIFDI AMDLGYVSQQ TFSRVFRRQF DRTPSDYRHR L	pJEC644
AsiA_m 2.1	ATGAACAAGAACATCGACACGGTCCGCGAAATC ATTACAGTAGCTTCCATTCTGATCAAGTTTTCCC GCGAGGATATCGTTGAAAACCGTGCGAACTTTA TTGCCTTCTTGAACGAAATTGGAGTTACTCACG AGGGGCGCAAGTTGAATCGGAACTCGTTTCGC AAGATTATAAGCAAACTGACGCAGGAAGACAAA AAAACTTTAATTGATGAGTTCAACGAGGGATTTG AGGGCGTTTATCGTTATTTGGAAATGTATACCAA TAAGTAA	MNKNIDTVREII TVASILIKFSRE DIVENRANFIA FLNEIGVTHEG RKLNRNSFRKI ISKLTQEDKKT LIDEFNEGFEG VYRYLEMYTN K	Addgene ID #158065

Supplementary Table 4. **SYNZIP sequences used in this study.** Plasmid sequences are constructed by introducing interaction module SYNZIP18 sequence in the activator region of the example dCas9 with N-terminal fusion, example dCas9 with C-terminal fusion, or in the SYNZIP region in dCas9-SYNZIP from **Supplementary Table 2**. Activator-SYNZIP plasmid contains the SYNZIP17 sequence.

Name	DNA sequence	Protein sequence	Plasmids
SYNZIP17	ATGAACGAAAAAGAAGAACTGAAATCTAA AAAAGCGGAACTGCGCAACCGCATCGAA CAGCTGAAACAGAAACGCGAACAGCTGA AACAGAAAATCGCGAACCTGCGCAAAGA AATCGAAGCGTATAAATAATGA	MNEKEELKSKKA ELRNRIEQLKQK REQLKQKIANLR KEIEAYK	pJEC626
SYNZIP18	ATGTCTATCGCGGCCACCCTGGAAAACG ACTTAGCACGCCTTGAGAATGAAAATGC TCGTCTGGAGAAAGATATCGCGAATCTC GAGCGTGACCTGGCAAAATTAGAGCGTG AGGAAGCTTATTTC	MSIAATLENDLAR LENENARLEKDIA NLERDLAKLERE EAYF	pJEC556, pJEC585, pJEC578, pJEC570, pJEC605, pJEC647-pJEC668

Supplementary Table 5. Covalent linker sequences used in this study. Plasmid sequences can be constructed by replacing the red region in the dCas9 fusions and Activator-SYNZIP example plasmids from **Supplementary Table 2** with the following linkers which fuse the dCas9 to an activator domain or SYNZIP18 protein, or the SYNZIP17 to an activator domain.

Name	DNA sequence	Protein sequence	Plasmids
Two alanine	GCTGCA/GCAGCT	AA	pJEC547- pJEC552, pJEC644, pJEC645, pJEC646
XTEN	AGTGGCTCAGAGACGCCGGGTACT TCTGAGTCCGCTACGCCTGAGTCT	SGSETPGTS ESATPES	pJEC629
Linker between SYNZIPs	GGAGGTTCTGGAGGTGGTGGGTCC	GGSGGGGS	pJEC556, pJEC585, pJEC578, pJEC570, pJEC626, pJEC605, pJEC647-pJEC668
Linker for AsiA	TGCGCAGGTGGTGGCGGTTCTGGT GGTGGTGGCAGC	CAGGGGSG GGGS	Addgene ID #158065

Supplementary Table 6. Bacterial αNTD sequences. Plasmid sequences can be constructed by replacing the green αNTD sequence in the Activator-SYNZIP plasmid map in **Supplementary Table 2** with the following αNTD sequences amplified from different bacterial genomes.

Strain source	DNA sequence	Protein sequence	Plasmids
Pseudomonas aeruginosa	ATGCAGAGTTCGGTAAATGAGTTCCTGAC CCCCCGCCACATCGATGTGCAGGTGGTC AGTCAAACCCGCGCCAAGATCACGCTCG AGCCTCTCGAGCGTGGTTTTGGTCACACC CTGGGCAACGCGCTGCGTCGCATCCTGT TGTCCTCCATGCCTGGCTGCGCAGTGGT CGAGGCCGAGATCGACGGCGTACTCCAC GAGTACTCGGCGATCGAAGGTGTGCAGG AAGATGTAATCGAGATCCTGCTGAACCTG AAAGGTCTGGCCATCAAGCTGCACGGTC GTGATGAAGTGACGCTGACCCTGGCTAA GAAGGGCTCGGGTGTTGTGACTGCTGCC GATATTCAGCTGGATCACGATGTTGAGAT CATCAACGGTGACCACGTTATCGCCAACC TGGCAGACAACGGCGCGCTGAACATGAA GCTGAAGGTAGCTCGTGGCCGTGGCTAC GAGCCTGCCGACGCACGTCAGAGCGATG AAGACGAAAGCCGCAGCATCGGCCGTCT GCAGCTCGACGCATCGTTCAGCCCGGTC CGTCGTGTCTCCTACGTGGTGGAAAACG	MQSSVNEFLT PRHIDVQVVS QTRAKITLEPL ERGFGHTLG NALRRILLSS MPGCAVVEA EIDGVLHEYS AIEGVQEDVI EILLNLKGLAI KLHGRDEVTL TLAKKGSGVV TAADIQLDHD VEIINGDHVIA NLADNGALN MKLKVARGR GYEPADARQ SDEDESRSIG RLQLDASFSP VRRVSYVVE NARVEQRTN	pJEC637

	CCCGTGTCGAGCAGCGCACCAACCTGGA<br>CAAACTGGTCCTGGACCTGGAAACCAAC<br>GGCACTCTGGATCCCGAAGAGGCTATCC<br>GTCGCGCCGCTACCATCCTGCAACAGCA<br>GCTGGCAGCGTTCGTGGACCTCAAG	LDKLVLDLET<br>NGTLDPEEAI<br>RRAATILQQQ<br>LAAFVDLK	
Pseudomonas fluorescens	ATGCAGATTTCGGTAAATGAGTTCCTGAC<br>ACCCCGCCACATTGATGTGCAGGTTGTCA<br>GTCCAACCCGCGCCAAAATTACTCTCGAG<br>CCTCTCGAGCGTGGTTTTGGCCACACCCT<br>GGGCAACGCGCTGCGCCGCATCCTGTTG<br>TCCTCAATGCCCGGCTGTGCAGTAGTCG<br>AGGCCGAGATTGACGGTGTGCTCCACGA<br>GTACAGCGCCATCGAAGGTGTACAGGAA<br>GACGTAATTGAAATCCTGTTGAACCTTAA<br>AGGTCTGGCTATCAAGCTGCACGGCCGT<br>GACGAAGTTACGCTGACCTTGTCGAAGAA<br>GGGTTCGGGGGTGGTTACCGCTGCCGAT<br>ATTCAGCTGGATCATGATGTCGAGATCGT<br>TAACCCCGATCACGTAATCGCTAACCTGG<br>CGTCTAACGGCGCCCTGAACATGAAGCT<br>CACTGTAGCTCGTGGTCGTGGTTATGAAC<br>CGGCCGACTCGCGTCAGAGCGATGAAGA<br>CGAAAGCCGCAGCATTGGTCGCTTGCAG<br>CTTGACTCTTCGTTCAGCCCGGTTCGCCG<br>TATCGCATACGTGGTGGAAAACGCCCGT<br>GTCGAGCAGCGTACTAACCTGGACAAGC<br>TGGTTATTGATCTGGAAACCAACGGTACC<br>CTGGATCCTGAAGAGGCTATCCGCCGCG<br>CTGCAACCATTCTGCAACAGCAGTTGGCT<br>GCGTTCGTCGACCTCAAG	MQISVNEFLT<br>PRHIDVQVVS<br>PTRAKITLEPL<br>ERGFGHTLG<br>NALRRILLSS<br>MPGCAVVEA<br>EIDGVLHEYS<br>AIEGVQEDVI<br>EILLNLKGLAI<br>KLHGRDEVTL<br>TLSKKGSGVV<br>TAADIQLDHD<br>VEIVNPDHVIA<br>NLASNGALN<br>MKLTVARGR<br>GYEPADSRQ<br>SDEDESRSIG<br>RLQLDSSFSP<br>VRRIAYVVEN<br>ARVEQRTNL<br>DKLVIDLETN<br>GTLDPEEAIR<br>RAATILQQQL<br>AAFVDLK	pJEC638
Pseudomonas stutzeri	ATGCAGAGTTCGGTAAATGAGTTCCTGAC<br>CCCCCGCCATATCGATGTGCAGGTGGTC<br>AGTCCGACCCGTGCCAAGATCACGCTCG<br>AGCCTCTCGAGCGCGGTTTCGGCCACAC<br>CCTGGGCAACGCGCTGCGTCGCATTCTG<br>TTGTCCTCCATGCCTGGCTGCGCCGTGG<br>TCGAGGCTGAGATCGACGGCGTGCTCCA<br>CGAGTACAGCGCCATCGAGGGCGTGCAG<br>GAAGATGTCATCGAAATCCTGCTCAACCT<br>CAAAGGTATCGCCATCAAGCTGCACGGC<br>CGTGATGAAGTGACCTTGAGCCTGGTGA<br>AGAAGGGCGCGGGCCCTGTTACCGCTGC<br>CGATATCCAGCTGGATCACGATGTCGAAA<br>TCGTCAATGGCGATCACCTGATCGCCAAC<br>CTGGCGGCCAATGGCTCGATCAACATGA<br>AGCTCAAGGTCGCTCGCGGCCGTGGTTA<br>CGAGCCGGCTGATGCGCGTCAGAGCGAC<br>GAGGATGAAAGCCGCAGTATCGGCCGTC<br>TTCAGCTCGACGCCACTTTCAGCCCGGT<br>GCGTCGTGTGGCTTATGTGGTCGAGAAC<br>GCTCGTGTCGAACAGCGCACCAACCTGG	MQSSVNEFLT<br>PRHIDVQVVS<br>PTRAKITLEPL<br>ERGFGHTLG<br>NALRRILLSS<br>MPGCAVVEA<br>EIDGVLHEYS<br>AIEGVQEDVI<br>EILLNLKGIAIK<br>LHGRDEVTLS<br>LVKKGAGPVT<br>AADIQLDHDV<br>EIVNGDHLIAN<br>LAANGSINMK<br>LKVARGRGY<br>EPADARQSD<br>EDESRSIGRL<br>QLDATFSPVR<br>RVAYVVENA<br>RVEQRTNLD<br>KLVIDLETNG	pJEC639

	ACAAGCTGGTTATCGATCTCGAAACCAAC<br>GGCACCCTGGATCCCGAAGAGGCGATCC<br>GTCGCGCCGCGACTATCCTGCAACAGCA<br>ACTGGCTGCGTTCGTCGACCTGAAG	TLDPEEAIRR<br>AATILQQQLA<br>AFVDLK	
Ruegeria pomeroyi	ATGATCCACAAGAACTGGGCAGAACTGAT<br>CAAGCCGACCCAGCTTGAGGTCAAGCCG<br>GGCAACGACCCGGCACGCCAGGCGACC<br>GTGATCGCCGAACCGCTGGAGCGTGGTT<br>TCGGTCTGACGCTGGGCAACGCTCTGCG<br>CCGCATCCTGATGAGCTCGCTGCAAGGC<br>GCGGCCATCAGCAGCGTGCAGATCGACA<br>ACGTCCTGCACGAGTTTTCGAGCGTGGC<br>CGGTGTGCGCGAAGACGTCACCGACATC<br>ATCCTGAACCTCAAGCAGGTCTCGCTTCG<br>CATGGATGTCGAAGGGCCCAAGCGCCTG<br>TCGGTCAATGCCAAGGGCCCGGCCGTCG<br>TCACCGCCGGTGACATCAGCGAAAGCGC<br>CGGCATCGAGGTGCTGAACCGCGACCAC<br>GTGATCTGTCACCTGGACGATGGCGCCG<br>ACCTGTTCATGGAACTGACCGTGAATACC<br>GGCAAGGGTTATGTGTCGGCCGACAAGA<br>ACCGCCCCGAAGACGCGCCCATCGGCCT<br>GATCCCGATCGACGCGATCTATTCGCCG<br>GTGAAAAAGGTCGCCTATGACGTGCAGC<br>CCACCCGCGAGGGCCAGGTTTTGGACTA<br>TGACAAGCTGACCATGAAGATCGAGACC<br>GACGGCTCGATCACCCCGGACGATGCCG<br>TGGCCTTTGCTGCGCGTATCCTTCAGGAT<br>CAGCTGTCGATCTTCGTCAACTTCGAC	MIHKNWAELI<br>KPTQLEVKPG<br>NDPARQATVI<br>AEPLERGFGL<br>TLGNALRRIL<br>MSSLQGAAIS<br>SVQIDNVLHE<br>FSSVAGVRE<br>DVTDIILNLKQ<br>VSLRMDVEG<br>PKRLSVNAKG<br>PAVVTAGDIS<br>ESAGIEVLNR<br>DHVICHLDDG<br>ADLFMELTVN<br>TGKGYVSAD<br>KNRPEDAPIG<br>LIPIDAIYSPVK<br>KVAYDVQPT<br>REGQVLDYD<br>KLTMKIETDG<br>SITPDDAVAF<br>AARILQDQLSI<br>FVNFD	pJEC640
Ruegeria sp. TM1040	ATGATCCACAAGAATTGGGCAGAATTGAT<br>CAAGCCCACGCAGCTTGAGGTGAAACCG<br>GGCAATGATCCGGCACGTCAGGCAACGC<br>TCGTTGCGGAACCGCTGGAGCGTGGCTT<br>TGGTCTGACGCTCGGCAACGCGCTGCGC<br>CGCATCCTGATGAGCTCGCTGCAAGGCG<br>CGGCCATCACATCCGTCCAGATCGACAA<br>CGTGCTGCACGAGTTTTCCTCCGTGGCC<br>GGTGTTCGTGAAGACGTCACAGACATCAT<br>CCTGAACCTCAAGCAGGTCTCCCTGCGC<br>ATGGAAGTCGAAGGGCCCAAGCGCCTGT<br>CGATCAATGCCAAAGGTCCGGCCGTCGT<br>CACCGCAGGCGACATTGCCGAAACCGCT<br>GGCATCGAAGTTCTGAACCGCGAGCACG<br>TCATCTGCCACCTCGACGATGGTGCGGA<br>TCTGTTCATGGAACTCACTGTCAACACCG<br>GCAAAGGCTATGTCTCTGCCGAGAAGAA<br>CAAGCCCGAGGACGCACCGATTGGTCTT<br>ATTCCGATCGACGCGATCTATTCCCCGGT<br>CAAGAAGGTCTCTTACGACGTTCAGCCGA<br>CCCGCGAAGGTCAGGTTCTGGACTATGA<br>CAAGCTGACCCTCAAAGTTGACACCGAC	MIHKNWAELI<br>KPTQLEVKPG<br>NDPARQATLV<br>AEPLERGFGL<br>TLGNALRRIL<br>MSSLQGAAIT<br>SVQIDNVLHE<br>FSSVAGVRE<br>DVTDIILNLKQ<br>VSLRMEVEG<br>PKRLSINAKG<br>PAVVTAGDIA<br>ETAGIEVLNR<br>EHVICHLDDG<br>ADLFMELTVN<br>TGKGYVSAE<br>KNKPEDAPIG<br>LIPIDAIYSPVK<br>KVSYDVQPT<br>REGQVLDYD<br>KLTLKVDTDG<br>SITPEDALAFA	pJEC641

	GGCTCCATCACCCCCGAAGACGCGCTGG<br>CTTTTGCGGCCCGCATCCTTCAGGACCA<br>GCTGTCGATCTTCGTCAACTTCGAC	ARILQDQLSIF<br>VNFD	
Rhodobacter capsulatus	ATGATCCACAAGAATTGGGCCGAACTGAT<br>CAAGCCGACGCAGCTTGAAATCAAGGCG<br>GGCAACGATCCGCAACGGCAAGCCACCG<br>TCATCGCGGAACCGCTGGAACGCGGCTT<br>CGGTCTGACGCTGGGCAACGCGCTGCGC<br>CGCGTTCTGATGAGCTCGCTGCAAGGCG<br>CGGCGATCACCTCGGTGCAGATCGACAA<br>CGTCCTGCACGAGTTTTCGAGCGTCGCG<br>GGCGTGCGGGAAGACGTCACCGACATCA<br>TCCTGAACCTCAAGGGCATCTCGCTCAAG<br>ATGGATGTCGAAGGGCCGAAACGGCTCT<br>CGATCTCGGCCAAGGGTCCGCGCGTCGT<br>GACCGGCGCCGACATCTCGGAATCGATG<br>GGCATCGAGGTCCTGAACAAGGATCACG<br>TGATCTGCCACCTTGACGATGGCGCCGA<br>CGTGTTCATGGAGCTGACCGTGAACACC<br>GGCAAGGGCTATGTGGCCGCCGACAAGA<br>ACCGTCCGGAAGATGCGCCGATCGGGCT<br>GATCCCGATCGATGCGATCTATTCGCCG<br>GTGAAGAAGGTCGCCTATGACGTGCAAC<br>CGACCCGCGAGGGCCAGGTGCTCGACTA<br>TGACAAGCTGACGATGAAGATCGAGACT<br>GACGGTTCGCTGACGCCCGAGGACGCG<br>GTGGCCTATGCCGCCCGCATCCTGCAGG<br>ACCAGCTGTCGATCTTCGTGAACTTCGAA	MIHKNWAELI<br>KPTQLEIKAG<br>NDPQRQATVI<br>AEPLERGFGL<br>TLGNALRRVL<br>MSSLQGAAIT<br>SVQIDNVLHE<br>FSSVAGVRE<br>DVTDIILNLKG<br>ISLKMDVEGP<br>KRLSISAKGP<br>RVVTGADISE<br>SMGIEVLNKD<br>HVICHLDDGA<br>DVFMELTVNT<br>GKGYVAADK<br>NRPEDAPIGLI<br>PIDAIYSPVKK<br>VAYDVQPTR<br>EGQVLDYDKL<br>TMKIETDGSL<br>TPEDAVAYAA<br>RILQDQLSIFV<br>NFE	pJEC642
Shewanella oneidensis	ATGCAGGGTTCTGTTACAGAATTTCTTAAA<br>CCGCGTCTCGTTGATATCGAGCAGGTTAA<br>CTCAACACGTGCCAAGGTTACTTTGGAAC<br>CGCTTGAGCGTGGTTTCGGCCACACTTTA<br>GGTAACGCGTTGCGTCGCATCCTATTGTC<br>GTCTATGCCCGGCTGCGCGGTTACCGAA<br>GTCGAGATTGACGGCGTACTGCACGAAT<br>ACAGCAGTAAGGAAGGCGTACAAGAAGA<br>TATCCTTGAAATCTTGCTAAATCTGAAAGG<br>CTTAGCAGTGACTATCGAGGGTAAAGACG<br>AGGCTATGCTTACATTAAGCAAGTCCGGC<br>GCAGGCCCTGTCATCGCAGCAGATATCA<br>CGCATGATGGTGATGTCACTATCGTGAAT<br>CCTGATCATGTTATCTGTCATTTAACAGGT<br>AACAATGATATCAGCATGCGTATTCGCGT<br>TGAGCGTGGTCGTGGTTATGTGCCAGCA<br>TCTGCTCGTGCACAGACAGAAGACGATG<br>ATCGCCCAATCGGCCGTTTGCTGGTTGAT<br>GCTTCTTTCTCGCCAGTCGCACGTATTGC<br>CTACAATGTAGAAGCAGCTCGTGTTGAAC<br>AGCGTACTGACTTGGATAAACTCGTTATC<br>GATATGACCACTAACGGTACTATTGATCC<br>TGAGGAAGCTATCCGTCGTTCTGCAACCA	MQGSVTEFL<br>KPRLVDIEQV<br>NSTRAKVTLE<br>PLERGFGHTL<br>GNALRRILLS<br>SMPGCAVTE<br>VEIDGVLHEY<br>SSKEGVQEDI<br>LEILLNLKGLA<br>VTIEGKDEAM<br>LTLSKSGAGP<br>VIAADITHDG<br>DVTIVNPDHVI<br>CHLTGNNDIS<br>MRIRVERGR<br>GYVPASARA<br>QTEDDDRPIG<br>RLLVDASFSP<br>VARIAYNVEA<br>ARVEQRTDL<br>DKLVIDMTTN<br>GTIDPEEAIRR	pJEC643

	TCTTAGCTGAACAGCTGGATGCGTTTGTC GAGCTGCGT	SATILAEQLD AFVELR	

Supplementary Table 7. sgRNA sequences used in this study. Plasmids were constructed by replacing the sgRNA spacer region with the following sequences.

Plasmid	Targeting position*	Spacer	Name
pJEC608	-70T	CGGTGTCCTGCGGTTACCAA	sgRNA 70T
pJEC567	-80T	AGGTATCCTGCGGTGTCCTG	sgRNA 80T
pJEC609	-90T	GGGCGACCTCAGGTATCCTG	sgRNA 90T
pJEC583	-100T	GGGCCACCACGGGCGACCTC	sgRNA 100T
pJEC564	-61NT	AGGTATCCGGTGGAACCCAA	sgRNA 61NT
pJEC610	-71NT	TGGAACCCAAAGGACGCCTT	sgRNA 71NT
pJEC566	-81NT	AGGACGCCTTTGGTAACCGC	sgRNA 81NT
pJEC611	-91NT	TGGTAACCGCAGGACACCGC	sgRNA 91NT
pJEC584	-121NT	AGGTCGCCCGTGGTGGCCCA	sgRNA 121NT

*Targeting position is calculated from the nucleotide position between the PAM and the complementary sequence to the TSS for reporter plasmid pJEC581.

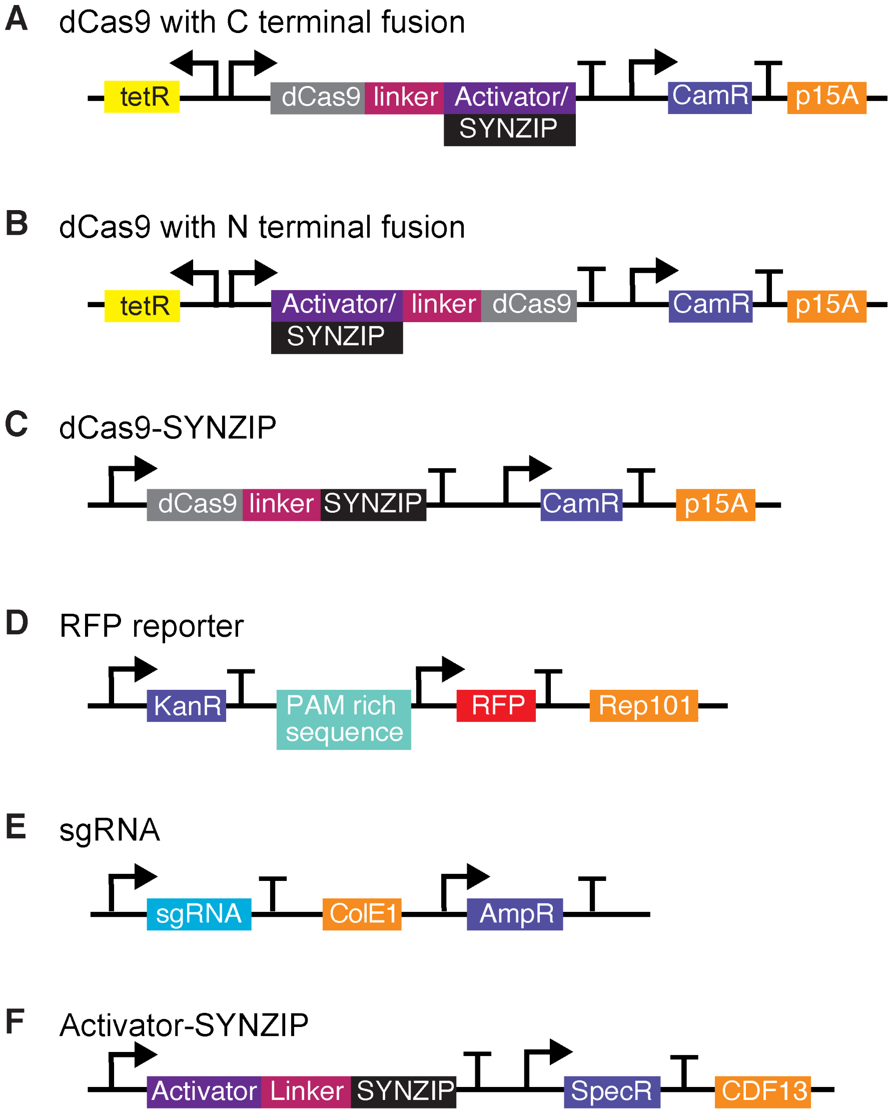

Supplementary Figure 1. Maps of DNA plasmids used in this study. dCas9 with C-terminal (**a**) or N-terminal fusion (**b**) to an AD or SYNZIP under the control of an anhydrotetracycline (aTc) inducible promoter. (**c**) dCas9 fused to SYNZIP18. (**d**) RFP reporter containing a synthetic PAM rich sequence derived from addgene plasmid ID #113322 (1, 2) upstream of the promoter with sgRNA binding sites every 10 bp. Derived reporter plasmids contain additional nucleotides between the PAM rich sequence and the RFP promoter. (**e**) sgRNA plasmids targeting different sequences upstream of the promoter of the RFP reporter. (**f**) AD fused to SYNZIP17. p15A, Rep101, ColE1 and CDF are origins of replication. CamR, KanR, AmpR and SpecR are Chloramphenicol, Kanamycin, Ampicillin and Spectinomycin resistance genes respectively.

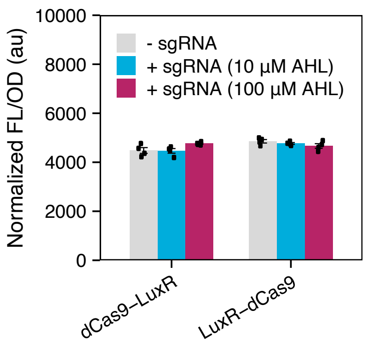

Supplementary Figure 2. Evaluation of activation with a LuxR AD in the presence of N-acyl homoserine lactone (AHL). Fluorescence characterization of LuxR AD in the presence of 1 ng mL^{-1} of aTc and in the presence (10 and 100 µM) and absence of its cognate inducer, AHL. Fluorescence characterization (measured in units of fluorescence [FL]/optical density [OD] at 600 nm) was performed with *E. coli* cells transformed with an RFP reporter plasmid, a plasmid encoding the dCas9 and LuxR fusions, and a sgRNA-encoding plasmid (+ sgRNA) or a no-sgRNA control plasmid (- sgRNA). sgRNA variant used targeted a PAM located at 81 bp upstream of the promoter TSS on the non-template strand. FL/OD values were normalized against blank cells. Data represent mean values and error bars represent s.d. of n = 4 biological replicates. P value > 0.05 for all + sgRNA conditions compared with the no-sgRNA control.

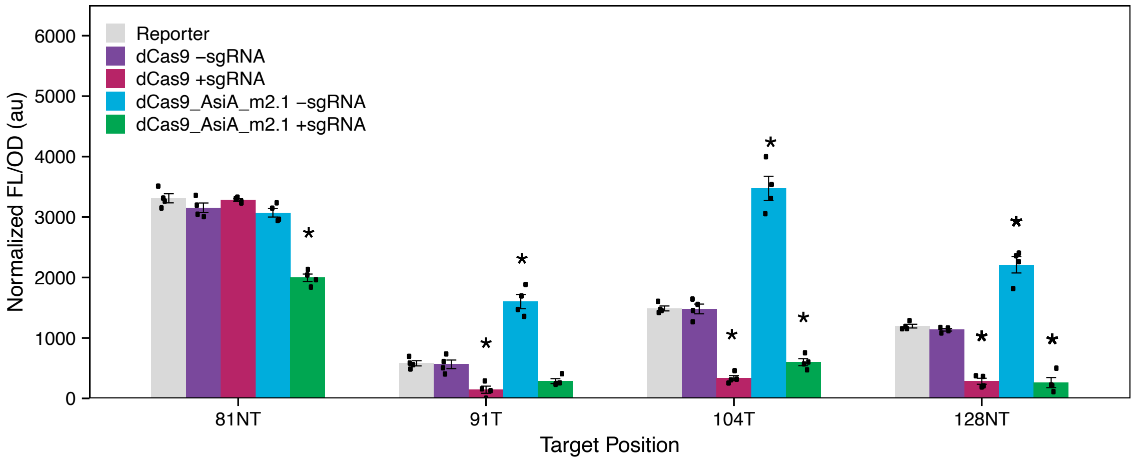

Supplementary Figure 3. Evaluation of AsiAm2.1 as an AD. Fluorescence characterization of a reported CRISPRa system (addgene ID #158065) in which the dCas9 is fused to an evolved variant of the AsiA protein called AsiA_m2.1 through a CAGGGGSGGGGS linker (3). Fluorescence characterization (measured in units of fluorescence [FL]/optical density [OD] at 600 nm) was performed with *E. coli* cells transformed with RFP reporter plasmids, a plasmid encoding dCas9_ AsiA_m2.1 fusion or a control dCas9 plasmid, and a sgRNA-encoding plasmid (+ sgRNA) or a no-sgRNA control plasmid (- sgRNA). sgRNAs targeted PAM sites in positions 81NT (sgRNA plasmid pJEC566 and reporter plasmid pJEC581), 91T (sgRNA plasmid pJEC609 and reporter pJEC597), 104T (sgRNA plasmid pJEC583 and reporter pJEC594), and 128NT (sgRNA plasmid pJEC584 and reporter pJEC591). Additionally a reporter control expressing only the reporter plasmid with no dCas9 or sgRNA was included for each condition. FL/OD values were normalized against blank cells. Data represent mean values and error bars represent s.d. of $n = 4$ biological replicates. Asterisks indicate P value comparing each condition with the Reporter control *P<0.001; P>0.001 has no asterisk.

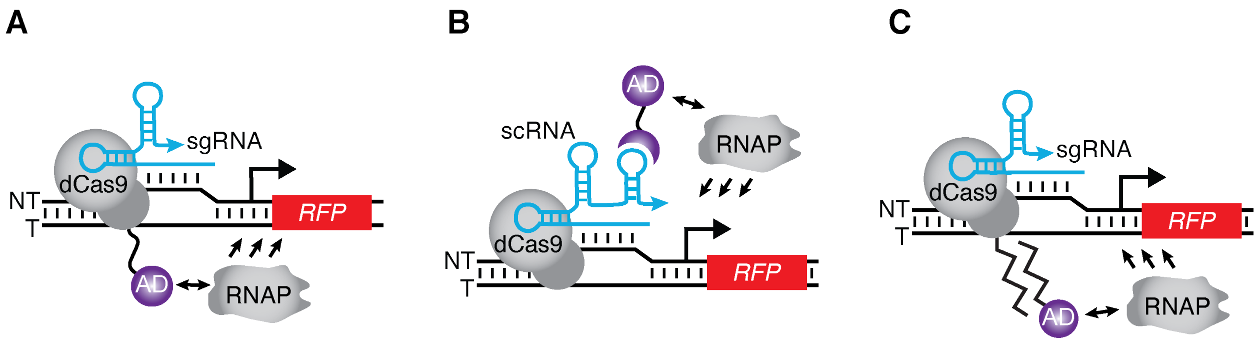

Supplementary Figure 4. Schematic of bacterial CRISPRa systems with different AD recruitment strategies. Bacterial CRISPRa localize an AD upstream of a gene of interest, recruiting the RNAP to the promoter to initiate transcription. (**a**) Direct fusion of the AD to the dCas9 through a covalent linker (3, 4). (**b**) Engineering of the sgRNA to generate a scaffold RNA that contains an RNA hairpin. This RNA hairpin is specifically recognized by the MS2 coat protein that is covalently fused to an AD (1). (**c**) Modular CRISPRa approach described here, in which the dCas9 and AD are independently fused to SYNZIP protein domains that form heterodimers through noncovalent interactions.

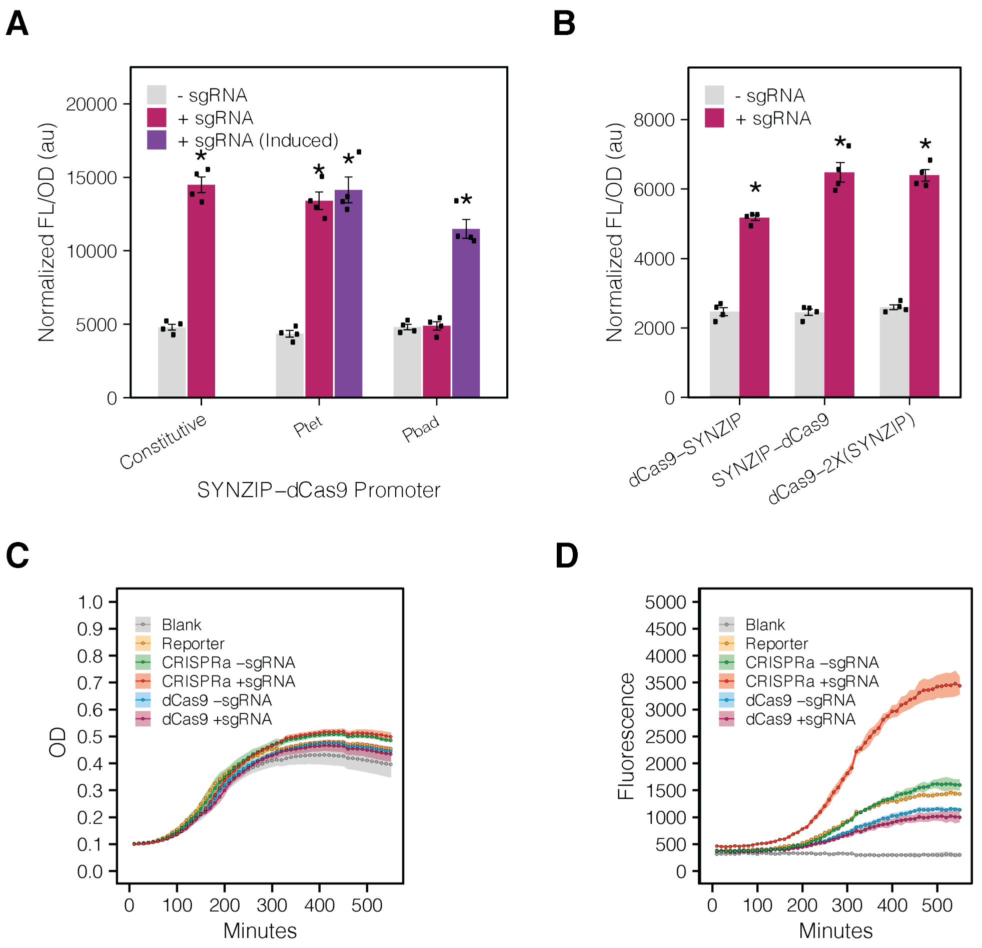

Supplementary Figure 5. Characterization of SYNZIP fusions to dCas9. (**a**) Fluorescence characterization of CRISPRa with SYNZIP-dCas9 under the control of a constitutive promoter J23150, or inducible promoters P_{tet} and P_{bad}. Induction was performed with a final concentration of 1 ng mL^{-1} of aTc for the P_{tet} promoter and 50 mM of arabinose for the P_{bad} promoter. (**b**) Fluorescence characterization of different designs for dCas9 fusions to SYNZIP18 under the control of a constitutive promoter. We evaluated the SYNZIP18 fused to the C-terminus (dCas9-SYNZIP), to the N-terminus (SYNZIP-dCas9) as well as the presence of two consecutive SYNZIP18 fused to the C-terminus (dCas9-2X(SYNZIP)). Time course evaluation of growth (**c**) in units of optical density at 600 nm and FL (**d**) in arbitrary units was performed for *E. coli* cells with different conditions: Blank cells expressing only antibiotic resistance, Reporter cells expressing the RFP reporter plasmid, and cells expressing the RFP reporter and dCas9 or the modular CRISPRa system in the presence or absence of a sgRNA. Measurements were taken every 10 minutes for up to 10h. Fluorescence characterization (measured in units of fluorescence [FL]/optical density [OD] at 600 nm) was performed with *E. coli* cells transformed with an RFP reporter plasmid, a plasmid encoding αNTD-SYNZIP, a plasmid encoding the dCas9 fusions to SYNZIP or control dCas9, and a plasmid encoding a sgRNA variant targeting a PAM located at 81 bp upstream of the promoter TSS on the non-template strand (+ sgRNA) or a no-sgRNA control plasmid (- sgRNA). Additionally a control expressing only the reporter plasmid with no dCas9 and no sgRNA was included for the time course evaluations. FL/OD values were normalized against blank cells. Data represent mean values and error bars or shading represent s.d. of n = 4 biological replicates. Asterisks indicate P value comparing each condition with the no-sgRNA control *P<0.0001.

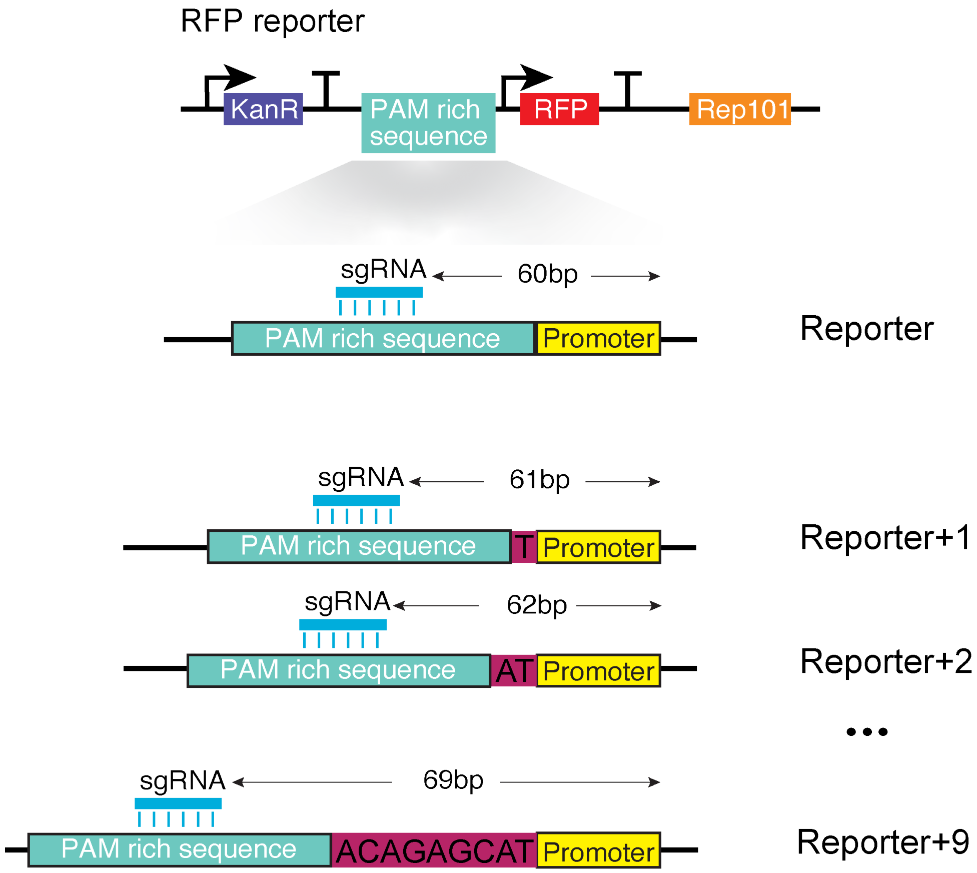

Supplementary Figure 6. Reporter plasmids used to characterize activation patterns at nucleotide resolution. We adopted a strategy previously described (2) to create reporter plasmids that allowed us to characterize CRISPRa activation patterns at nucleotide resolution. Initial reporter sequence contains a protospacer adjacent motif (PAM) rich sequence, where NGG nucleotides are present every 10 bp in the template (T) and non-template (NT) strand in the sequence upstream of the promoter. sgRNA-encoding plasmids were designed to target each of these PAM sites. To allow characterization of binding sites in between, 1-9 nucleotides were inserted between the promoter and the upstream PAM rich sequence, generating nine additional reporters (Reporter +1 to Reporter +9) that had shifted distances between the position targeted by the sgRNAs and the promoter TSS. For example, a sgRNA targeting a position 60 bp upstream of the TSS on the template strand (60T) in the original reporter plasmid can be used to characterize positions from 61 T to 69 T (as shown above) when the corresponding reporter plasmids are used. Using this strategy, we used 7 sgRNAs to characterize activation patterns between positions 60 and 100 in the T and NT strand.

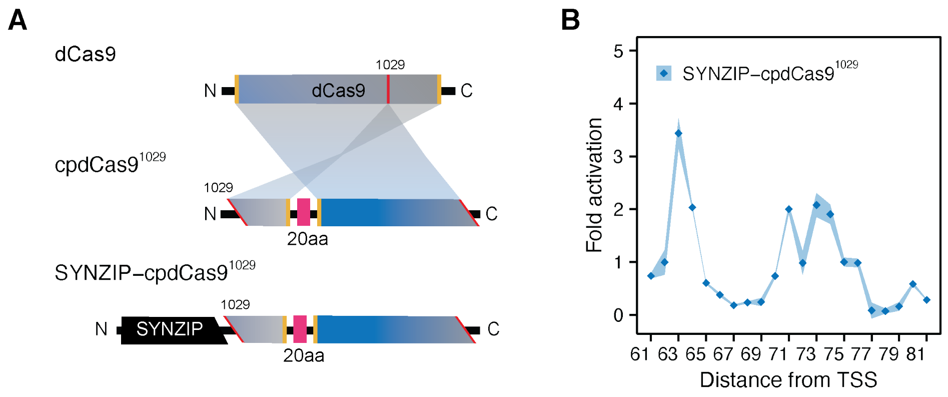

Supplementary Figure 7. Characterization of activation with SYNZIP-cpdCas9^{1029}. (**a**) Cloning of SYNZIP-cpdCas9^{1029}. The cpdCas9^{1029} variant was selected from a library of reported cpdCas9 variants (5), where the original N- and C-termini of the dCas9 (yellow) are fused with a 20 amino acid linker (20aa) and new N- and C-termini are created at former residue position 1029 (red). SYNZIP18 was fused to the N-terminus of cpdCas9^{1029} to incorporate the cpdCas9 into our modular CRISPRa system. (**b**) Characterization of the distance-dependent activation patterns of CRISPRa with cpdCas9^{1029} was evaluated by targeting the system to positions located between 61 to 81 bp upstream of the promoter in the non-template strand. Fluorescence characterization (measured in units of fluorescence [FL]/optical density [OD] at 600 nm) was performed in MG1655 *E. coli* cells transformed with an RFP reporter plasmid, a plasmid encoding the cpdCas9^{1029}, a sgRNA plasmid or a no-sgRNA control plasmid, and a plasmid encoding αNTD-SYNZIP. FL/OD values were normalized against blank cells. Fold activation was calculated by dividing the FL/OD obtained in the presence of a targeting sgRNA with the FL/OD obtained using a no-sgRNA control for each reporter plasmid. Data represent mean values and shading represent s.d. of n = 4 biological replicates.

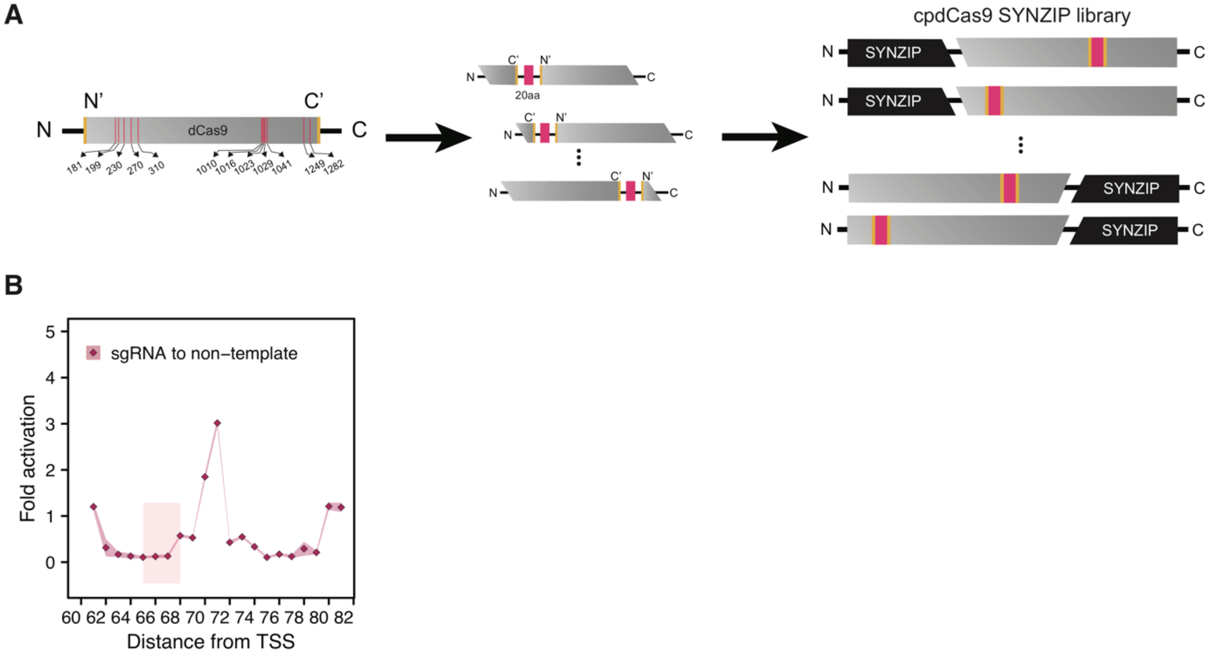

Supplementary Figure 8. Screening strategy to identify activating cpdCas9 variants. (**a**) Eleven circularly permutated dCas9 variants were cloned fusing the original N- and C-terminal domains (N' and C') with a 20 amino acid linker and creating new N- and C-termini from 11 different residue positions. SYNZIP18 was fused to the new N- or C-terminus of cpdCas9 variants, generating a library of 22 variants. (**b**) Characterization of distance-dependent activation patterns of dCas9-SYNZIP targeted to the RFP reporter between positions 61 and 100 bp upstream of the TSS on the non-template strand. Screening with the library of circularly permutated dCas9 variants was performed in the area indicated on the graph (shaded box), where no activation was achieved, corresponding to binding positions 65 to 68 upstream of the TSS on the non-template strand.

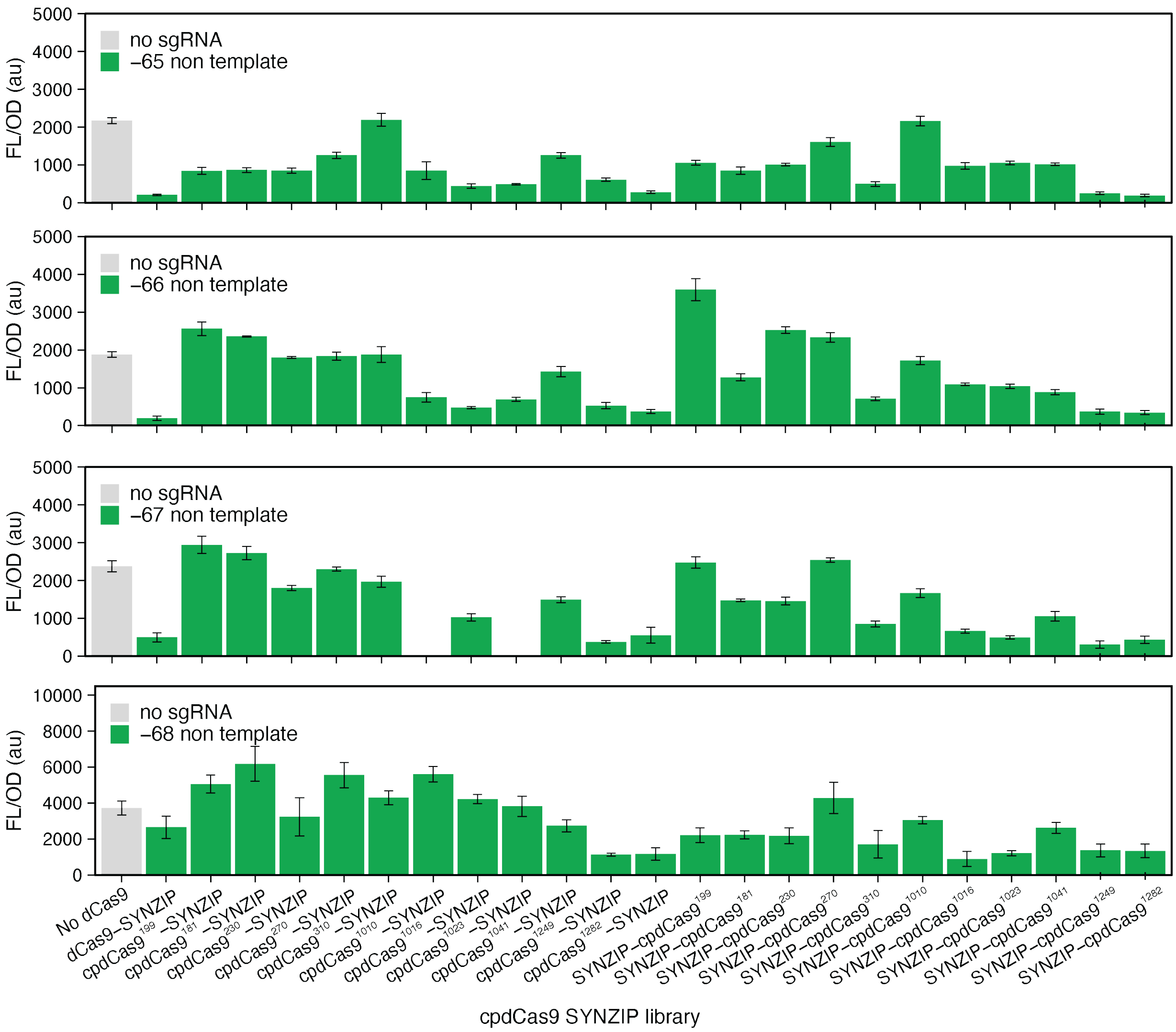

Supplementary Figure 9. Screening of activation with the cpdCas9 library targeting the non-template strand. Fluorescence characterization was performed in *E. coli* strain MG1655 transformed with the plasmid encoding the αNTD-SYNZIP, a plasmid encoding a sgRNA or no-sgRNA control, the RFP reporter plasmid, and a no-dCas9 control, dCas9-encoding plasmid or the library of circularly permutated dCas9 variants. Fluorescence characterization was performed by bulk fluorescence measurements (measured in units of fluorescence [FL]/optical density [OD] at 600 nm). FL/OD values were normalized against blank cells. Each graph is characterization data with a different reporter to measure activation at different binding positions between 65 to 68 bp upstream of the TSS on the non-template strand, compared to cells containing a no-sgRNA control (no sgRNA). Error bars represent the s.d. of biological replicates; areas missing a bar were not evaluated.

References

1. Dong,C., Fontana,J., Patel,A., Carothers,J.M. and Zalatan,J.G. (2018) Synthetic CRISPR-Cas gene activators for transcriptional reprogramming in bacteria. *Nat. Commun.*, **9**, 2489.

2. Fontana,J., Dong,C., Kiattisewee,C., Chavali,V.P., Tickman,B.I., Carothers,J.M. and Zalatan,J.G. (2020) Effective CRISPRa-mediated control of gene expression in bacteria must overcome strict target site requirements. *Nat. Commun.*, **11**, 1618.

3. Ho,H., Fang,J.R., Cheung,J. and Wang,H.H. (2020) Programmable CRISPR-Cas transcriptional activation in bacteria. *Mol. Syst. Biol.*, **16**, e9427.

4. Bikard,D., Jiang,W., Samai,P., Hochschild,A., Zhang,F. and Marraffini,L.A. (2013) Programmable repression and activation of bacterial gene expression using an engineered CRISPR-Cas system. *Nucleic Acids Res.*, **41**, 7429–7437.

5. Oakes,B.L., Fellmann,C., Rishi,H., Taylor,K.L., Ren,S.M., Nadler,D.C., Yokoo,R., Arkin,A.P., Doudna,J.A. and Savage,D.F. (2019) CRISPR-Cas9 Circular Permutants as Programmable Scaffolds for Genome Modification. *Cell*, **176**, 254-267.e16.
